# Flagellin/NLRC4 Pathway Rescues NLRP3-Inflammasome Defect in Dendritic Cells From HIV-Infected Patients: Perspective for New Adjuvant in Immunocompromised Individuals

**DOI:** 10.3389/fimmu.2019.01291

**Published:** 2019-06-11

**Authors:** Edione Cristina dos Reis, Vinícius Nunes Cordeiro Leal, Jaíne Lima da Silva Soares, Fernanda Pereira Fernandes, Dhêmerson Souza de Lima, Bruna Cunha de Alencar, Alessandra Pontillo

**Affiliations:** ^1^Laboratory of Immunogenetics, Department of Immunology, Institute of Biomedical Sciences/ICB, University of São Paulo/USP, São Paulo, Brazil; ^2^Laboratory of Immune System Cell Biology, Department of Immunology, Institute of Biomedical Sciences/ICB, University of São Paulo/USP, São Paulo, Brazil

**Keywords:** dendritic cell, inflammasome, HIV, flagellin, NLRC4, adjuvant

## Abstract

**Introduction:** NLRP3 inflammasome plays a key role in dendritic cells (DC) activation in response to vaccine adjuvants, however we previously showed that it is not properly activated in DC from HIV-infected patients (HIV-DC), explaining, at least in part, the poor response to immunization of these patients. Taking in account that several cytoplasmic receptors are able to activate inflammasome, and that bacterial components are considered as a novel and efficient adjuvant, we postulated that bacterial flagellin (FLG), a natural ligand of NAIP/NLRC4 inflammasome, could rescue the activation of the complex in HIV-DC.

**Objective:** Demonstrate that FLG is able to activate monocyte-derived dendritic cells from HIV-infected individuals better than LPS, and to what extent the entity of inflammasome activation differs between DC from HIV-infected patients and healthy donors.

**Methods:** Monocyte-derived dendritic cells from HIV-infected patients (HIV-DC) and healthy donors (HD-DC) were stimulated with FLG, and inflammasome as well as DC activation (phenotypic profile, cytokine production, autologous lymphocytes activation) were compared. Chemical and genetic inhibitors were used to depict the relative contribution of NLRC4 and NLRP3 in HIV/HD-DC response to FLG.

**Results:** FLG properly activates HD-DC and HIV-DC. FLG induces higher inflammasome activation than LPS in HIV-DC. FLG acts through NLRC4 and NLRP3 in HD-DC, but at a lesser extent in HIV-DC due to intrinsic NLRP3 defect.

**Conclusions:** FLG by-passes NLRP3 defect in HIV-DC, through the activation of NAIP/NLRC4 inflammasome, indicating possible future use of the bacterial component as an efficient adjuvant in immunocompromised individuals.

## Introduction

Dendritic cells (DC) are a specialized professional antigen presenting cells (APC) with unique capability to initiate and maintain primary immune responses when pulsed with antigens (1–3). Following recognition of pathogen- or damage-associated molecular patterns (PAMPs or DAMPs, respectively) by innate pattern recognition receptors (PRRs), DC activate and turn into a potent APC. The final differentiation of activated DC is characterized by plasma membrane up-regulation of MHC-II and co-stimulatory molecules (i.e., CD86, CD80), and the production of cytokines important for T CD4^+^ lymphocytes activation at the immunologic synapsis (i.e., IL-12 and IL-18; and/or IL-1ß; or IL-4). The result of DC activation drives the polarization of T CD4^+^ lymphocytes, and therefore of the immune response (4).

Successful vaccine preparations have to properly activate DC to induce a long-term memory protective immunity. Together with pathogen' antigens, adjuvants strongly contribute to the effectiveness of a vaccine. Their action is mediated by DC PRRs, such as Toll-like receptors (TLRs) and NACHT and LRR containing receptors (NLRs), through the activation of intracellular pathways leading to the production of cytokines important for T cell activation (5). Alum (aluminum hydroxide), a commonly used adjuvant, activates murine DC through the induction of the NLR-containing a PYD domain 3 (NLRP3) and the consequent mounting of the cytoplasmic complex, known as inflammasome, which results in caspase-1 activation and IL-1ß and IL-18 production. The absence of NLRP3 results in the loss of adjuvant responsivity (6), emphasizing the central role of inflammasome in the activation of DC and in the induction of an efficient immune response.

Accordingly, recent findings have reported that individuals with a low response to vaccines, such infants (7) or cancer patients (8) present a substantial alteration in inflammasome expression and/or activity.

Immune response to many current vaccines is known to be impaired and/or less effective in chronically HIV-infected individuals (9). This impairment has been associated to both a reduced frequency of DC (10, 11), together with phenotypic and functional alterations of these cells (12). As HIV-infected patients present well-documented DC impairment, it has been proposed that a poor response to vaccination could be caused by a diminished and/or defective response to common adjuvants (13).

We have previously demonstrated that NLRP3 inflammasome is not correctly activated by bacterial lipopolysaccharide (LPS) in monocyte-derived dendritic cells (MDDC) from HIV-infected patients (HIV-DC) (14), possibly as a result of the HIV-associated chronic inflammation and the consequent immune system exhaustion (15). As NLRP3 inflammasome is involved in the activation of DC by vaccine adjuvants (6, 16), the defect observed in NLRP3 inflammasome possibly contributes to the less extend immunization response in HIV-infected individuals.

To counteract the low immune response, new vaccination strategies have been proposed, such as the use of PRRs agonists, such as LPS or flagellin (FLG), as largely reviewed in (17).

FLG is the main component of a bacterial flagellum, and it is recognized extracellularly by TLR5 inducing a Myd88 signaling and promoting the transcription of NF-κB-related genes (18, 19); and by the intracellular receptors, NLR-containing a BIR domain (NAIP) and NLR-containing a CARD domain (NLRC)-4. NAIP directly binds FLG, while NLRC4 recognizes the NAIP:FLG complex and mounts the NAIP/NLRC4 inflammasome, resulting in IL-1ß and IL-18 production (20–22).

FLG has already been used as an adjuvant in a number of clinical trials of healthy individuals (23, 24), however to our knowledge none or poor data are available about its ability to activate MDDC in both healthy or immuno-compromised individuals via NAIP/NLRC4 inflammasome.

Taking in account the key role of the inflammasome in proper DC activation, and the impairment of NLRP3 activation observed in HIV-DC, we hypothesize that FLG could represent an alternative adjuvant for HIV-infected patients, by activating inflammasome in DC through NAIP and NLRC4 receptors, and in this way by-passing the NLRP3 defect. Therefore, the aim of this study was to demonstrate that FLG is able to activate MDDC from HIV-infected individuals better than LPS, and to what extent the entity of inflammasome activation differs between HIV-DC and HD-DC.

## Materials and Methods

### HIV-Infected Patients

Twenty-seven HIV-infected adults patients (16 males/11 females; 51.9 ± 11.7 years), proceeding from the metropolitan area of São Paulo (SP, Brazil), seropositive for at least 5 years (26.9 ± 16.9 years), in antiretroviral therapy (ART), with blood CD4^+^ T cells count >500 cells/μl, without clinical AIDS or other chronic diseases (i.e., neoplasias, cardiovascular disease, autoimmune disease, kidney disease, obesity) or infectious diseases (i.e., human T-lymphotropic virus/HTLV, hepatitis B or hepatitis C virus), were recruited from January 2016 to May 2018 at the “Serviço de Extensão ao Atendimento de Pacientes HIV/AIDS” (SEAP) of the Faculty of Medicine, University of São Paulo (São Paulo, SP, Brazil). Fifty milliliter of the peripheral blood was collected in heparin tubes. All volunteers assigned the informed consent approved by the Institutional Ethical Committee. Patients' main characteristics are summarized in Table 1.

**Table 1:** Main characteristics of healthy donors and HIV-infected patients.

	**HD (*n* = 27)**	**HIV (*n* = 27)**
Gender (M/F), n	15/12	16/11
Age (years), mean ± SD	45.5 ± 13.4	51.9 ± 11.7
Time from diagnosis (years), mean ± SD		26.9 ± 16.9
PVL^*^ (log RNA copies/μL), mean ± SD		1.77 ± 0.37
PVL^0^ (log RNA copies/μL), mean ± SD		4.00 ± 1.02
PVL^1^ (log RNA copies/μL), mean ± SD		2.25 ± 0.77
CD4^+^ T^*^ (cells/μL), mean ± SD		768.9 ± 283.1
CD4^+^ T^0^ (cells/μL), mean ± SD		377.5 ± 389.9
CD4^+^ T^1^ (cells/μL), mean ± SD		679.0 ± 252.7

Gender and age are reported for healthy donors (HD) and HIV-infected patients (HIV). Time from HIV-1 diagnosis as well as plasma viral load (PVL) and CD4^+^ T cells counts at the time of blood collection

(*) , before

(^0^) and after

(^1^) * the start of anti-retroviral therapy (ART) were included for HIV-infected patients. The detection limit of PVL was 1.70 log HIV-1 RNA copies/ml. M, males; F, females; n, number of individuals; SD, standard deviation*.

### Healthy Donors (HD)

Twenty-seven adults (15 males/12 females; 45.5 ± 13.4 years), proceeding from the metropolitan area of São Paulo (SP, Brazil), without clinical HIV or other chronic or infectious diseases, were recruited at the Blood Bank Service of the Hospital “Oswaldo Cruz” (São Paulo, SP, Brazil). Fifty milliliter of the peripheral blood was collected in heparin tubes. All volunteers assigned the informed consent approved by the Hospital Ethical Committee. HD demographic data were included in Table 1. Of note, any significant difference exists in gender ratio (Fisher test *p* > 0.05) or age mean value (*t*- test *p* > 0.05) between HD and HIV-infected patients.

### Human Monocyte-Derived Dendritic Cells (MDDC)

Mononuclear cells were isolated from 50 mL of peripheral blood by Ficoll-Hypaque (GE Healthcare) density gradient, and monocytes were separated from lymphocytes by plastic adherence. Briefly, 4 × 10^6^ mononuclear cells/well were incubated in 24-wells culture plates (Corning-Costar). After 2 h, non-adherent cells (mainly lymphocytes) were removed and cryopreserved at −80°C for co-culture assays, while adherent cells (mainly monocytes) were cultured with 50 ng/mL GM-CSF (Peprotech) and 50 ng/mL IL-4 (Peprotech) in RPMI-1640 (Gibco, Thermo Fisher Scientific) supplemented with 10% of fetal bovine serum (FBS; Gibco) at 37°C in 5% CO_2_ for 5 days (25). Monocytes-to-DC differentiation was confirmed by flow cytometry analysis of CD14 and CD11c surface markers (Supplementary File 1).

MDDC (HIV-DC or HD-DC) were stimulated with purified 5 μg/mL FLG from S. *typhimurium* (FLA-ST, Invivogen) or 1 μg/mL LPS from E. *coli* (Sigma-Aldrich, Merck) for 3, 8, 18, and 24 h and 1 mM ATP was added for more 15 min (26). In some experiments, MDDC were pre-incubated with 10 μg/mL MCC-950 (Invivogen), a specific NLRP3 inhibitor (27), or 10 μM parthenolide (PTD; Sigma-Aldrich, Merck), a NF-κB and caspases inhibitor (28); or with 1,000 UI/mL IFN-α-2b (Schering-Plough) for 18 h (29, 30). Cell supernatants were collected for cytokines measurement. MDDC were used for cytometric assays or RNA isolation and genes expression analysis.

To assess MDDC ability to activate CD4^+^ T lymphocytes, 0.4 × 10^5^ MDDC were distributed in 96-well U-bottom suspension culture plates with 4.0 × 10^5^ autologous lymphocytes (cryopreserved non-adherent peripheral blood mononuclear cells) (co-culture MDDC/lymphocytes ratio: 1/10) in duplicates and cultured in the presence of unspecific (not antigen-specific) stimuli for 96 or 120 h to measure IFN-γ production and lymphocytes proliferation, respectively. Lymphocytes alone were used as a negative control (Neg).

### MDDC Phenotype Analysis

2 × 10^5^ DC/mL were incubated in Phosphate Buffer Saline (PBS; Sigma-Aldrich, Merck) with the optimal dilution of anti-CD14 PE (MEM15; Exbio), anti-CD11c (3.9; Biolegend), anti-HLA-DR V500 (G46-6; BD Biosciences), anti-CD86 PE-cy7 (2331/FUN-1, B), and anti-CD40 Horizon 450 (5C3; BD Biosciences) antibodies for 20 min at 4°C. Cells were then washed twice with PBS and resuspended in 200 μL of 4% Formaldehyde-PBS. The Live/Dead Fixable Cell Stain Kit (Life Technology, Thermo Fisher Scientific) was added to the assay according to the manufacturer's instructions. A minimum of 50,000 events was acquired on a LSRFortessa™ X-20 flow cytometer (BD Biosciences) using the FACS Diva software (BD Biosciences). Data were analyzed using the FlowJo software (Tree Star). The gates strategy for one representative experiment was reported in Supplementary File 2.

### CD4^+^ T Lymphocytes Activation Assay

CD4^+^ T lymphocytes activation was measured by the meaning of intracellular staining of IFN-γ. Briefly, autologous co-cultures of MDDC and lymphocytes were treated with lymphocyte mitogens Ionomycin (1 μg/mL; Sigma-Aldrich, Merck) and Phorbol Myristate Acetate (10 ng/mL; Sigma-Aldrich, Merck) for 96 h (31). Twenty microgram per milliliter Brefeldin A (Sigma-Aldrich, Merck) was added 6 h before the end of co-culture to block Golgi secretory pathway. Cells were then labeled for surface marker anti-CD3 APC (MEM-57) and anti-CD4 PE (RPAT4) (BD Biosciences), permeabilized with Cytofix/Cytoperm solution (BD Biosciences), and finally stained for anti-IFN-γ V450 (B27; BD Biosciences). The Live/Dead Fixable Cell Stain Kit was added to the assay according to the manufacturer's instructions. Cells were then washed twice with PBS and resuspended in 200 μL of 4% Formaldehyde-PBS to proceed to flow cytometry analysis as above-mentioned.

### CD4^+^ T Lymphocytes Proliferation Assay

The CFSE Cell Division Tracker Kit (Biolegend) was used for flow cytometry analysis of *in vitro* CD4^+^ T cells proliferation assay according to manufacturers' protocol. Briefly, autologous lymphocytes were pre-treated with 0.1 μM CFSE before being added to MDDC cultures in the presence of the lymphocyte mitogen Concanavalin A (5 μg/mL; Sigma Aldrich, Merck) for 120 h (32). At the end of assay, cells were then labeled for anti-CD3 APC (MEM-57) and anti-CD4 PE (RPAT4) (BD Biosciences). The Live/Dead Fixable Cell Stain Kit was added to the assay according to the manufacturer's instructions. Cells were then washed twice with PBS and resuspended in 200 μL of 4% Formaldehyde-PBS to proceed to flow cytometry analysis as above-mentioned.

### Cytokines Measurement in Culture Supernatants

IL-1β, IL-18, TNF-α, and IL-12p70 were measured in MDDC culture supernatants by ELISA according to the manufacturers' protocols (Biolegend for IL-1β; IL-18, TNF; eBioscience for IL12p70). Data were reported as pg/mL.

### Caspase-1 Activity Assay

The detection of caspase-1 activity in MDDC was measured with the FAM FLICA Caspase-1 Assay Kit (Immunochemistry Technologies) and flow cytometry according to the manufacturer's protocol. Briefly, 10 μL 30x FLICA was added to 2 × 10^5^ MDDC in 300 mL and cells incubated for 1 h at 37°C 5% CO_2._ The Live/Dead Fixable Cell Stain Kit was used. Cells were then washed twice with PBS and resuspended in 200 μL of 4% Formaldehyde-PBS to proceed to flow cytometer analysis as above-mentioned. Live MDDC were gated based on their forward (FSC) and side light scatter (SSC). Histograms for one representative experiment was reported in Supplementary File 3.

### Inflammasome Genes Expression Analysis

Total RNA was isolated from 2 × 10^5^ MDDC using the RNAqueous-Micro kit (Ambion, Thermo Fisher Scientific) according to manufacturer's protocol and quantified using Nanodrop N-1000 (Agilent). 0.5 μg of total RNA was converted into cDNA using Superscript III RT kit and random primers (Invitrogen, Thermo Fisher Scientific). *NLRP1* (hs00248187), *NLRP3* (hs00366465), *NAIP* (hs03037952), *NLRC4* (hs00368367), *CASP1* (hs00354836), *IL1B* (hs01555410), *IL18* (hs01038788), *CARD8* (hs01088221), *BRCC3* (hs02386484), and *NEDD8* (hs01921826) genes were amplified using TaqMan® gene-specific assays (Applied Biosystems, Thermo Fisher Scientific) and qPCR on the QuantStudio 3.0 Real-Time PCR equipment (Applied Biosystems, Thermo Fisher Scientific). The QuantStudio 3.0 software was used to obtain cycle threshold values (Ct) for relative gene expression analysis according to Fold Change (FC) method (33). Raw expression data (Ct) were normalized with the expression of the housekeeping gene glyceraldehyde-3-phosphate dehydrogenase/*GAPDH* (hs02758991; TaqMan® assay) (ΔCt), and the FC was calculated comparing stimulated and unstimulated (UN) conditions (FC = 2^−ΔΔ*Ct*^; ΔΔCt = ΔCt_stimulated_-ΔCt_UN_). Alternatively, the basal (constitutive) gene expression was calculated as 2^−Δ*Ct*^.

### miR-223 Expression Analysis

Total RNA was isolated from 2 × 10^5^ MDDC by mirVana™ miRNA Isolation Kit (Ambion, Thermo Fisher Scientific) according to the manufacturer's instructions. 0.5 μg of total RNA were converted into cDNA using kit TaqMan™ MicroRNA Reverse Transcription and miRNA-specific primers (Applied Biosystems, Thermo Fisher Scientific). miR-223 was amplified using TaqMan® miR-specific assays (*TM:002098*; Applied Biosystems, Thermo Fisher Scientific) and qPCR on the QuantStudio 3.0 Real-Time PCR equipment. The QuantStudio 3.0 software was used to obtain cycle threshold values (Ct) for relative gene expression analysis according to Fold Change (FC) method (33). Raw expression data (Ct) were normalized with the expression of and non-coding small RNA control U6 (*TM:001973*; TaqMan® assay) (ΔCt), and the FC was calculated comparing stimulated and unstimulated (UN) conditions (FC = 2^−ΔΔ*Ct*^; ΔΔCt = ΔCt_stimulated_-ΔCt_UN_). Alternatively, the basal (constitutive) gene expression was calculated as 2^−Δ*Ct*^.

### Detection of “Specks” Formation

Detection of inflammasome “specks” formation was performed by confocal microscopy and immunofluorescence, as previously described (34). Briefly, 2 × 10^5^ MDDC were cultured in 16-wells chamber slides (Thermo Fisher Scientific) and stimulated with 5 μg/mL FLG or 1 μg/mL LPS for 24 h at 37°C 5% CO_2_ with or without 1 mM ATP. Cells were then fixed and permeabilized with Cytofix/Cytoperm reagent (BD Biosciences) for 30 min at 37°C 5% CO_2_, and incubated with primary antibody for NLRP3 (1:100 mouse anti-human NLRP3, Abcam) and/or NLRC4 (1:200 rabbit anti-human NLRC4; Biolegend) overnight at room temperature. Fluorescent secondary antibodies (Alexa 488-conjugated goat-anti-mouse IgG1, or Alexa 647-conjugated goat-anti-rabbit IgG1; Biolegend) were then added for 1 h. Finally, cells were washed and fixed to image acquisition at DMi8 confocal laser scanning microscope (Leica). 4′,6-Diamidine-2′-phenylindole dihydrochloride (DAPI; Sigma-Aldrich, Merck) was used for nuclear counterstaining. ImageJ software and related plugins (National Institutes of Health) were used for image processing. The counting of NLRP3+ and NLRC4+ specks in MDDC was performed manually by observing specks formation within the cells (34), and through the ImageJ software by calculating the corrected total cellular fluorescence (CTCF) for each marker as integrated density–(area of selected cell × mean fluorescence of background readings) (35).

### *NLRC4* and *NLRP3* Silencing

Pre-validated shRNA for human *NLRC4* and *NLRP3* was obtained from MISSION® shRNA Plasmid DNA (Sigma-Aldrich, Merck). The shRNAs for NLRP3 and NLRC4 used in the study are listed in Supplementary File 4. 2 × 10^5^ MDDC were transduced using with the same amounts of lentiviral particles encoding non-targeting control or gene-specific shRNA in the presence of SIV3+ VLP for 48 h. Thereafter, cells were treated with 5 μg/mL FLG or 1 μg/mL LPS for 24 h. The shRNA knockdown efficiency of the target protein in lentivirus-transduced cells was assessed by gene expression (Supplementary File 5). The concentrations of IL-1β in cell culture supernatants was measured by ELISA.

### Data Analysis

All data were collected and analyzed from at least three independent experiments. Normality test was applied to the data, and parametric or non-parametric analysis was used accordingly to compare two or more data sets as specified for each graph. The level of significance was *p* < 0.05. Calculations were performed using the statistical software package GraphPad Prism 7.0.

### Biosecurity and Institutional Safety Procedures

All research was performed following the guidelines of biosecurity and safety of Institute of Biomedical Science (ICB/USP).

## Results

### Flagellin Similarly Activates MDDC From HIV-Infected Patients and Healthy Donors

MDDC were treated with 5 μg/mL FLG for 24 h and phenotypic profile, TNF and IL-12 secretion as well as CD4^+^ T lymphocytes activation in co-culture experiments were assessed based on previously published protocols (31, 32).

**Figure 1:**
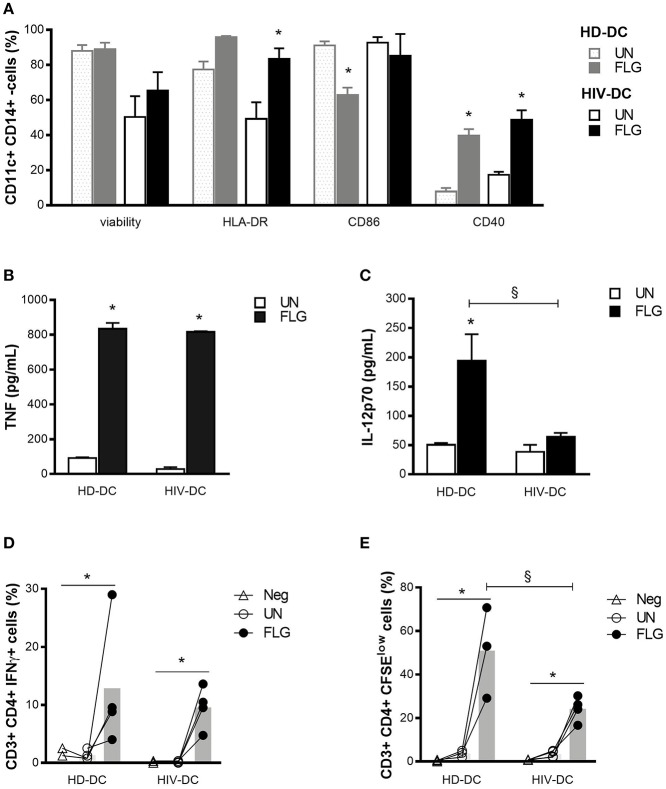
Flagellin similarly activates HIV-DC and HD-DC. 2 × 10^5^ MDDC from healthy donors (HD-DC; *n* = 5) and HIV-infected patients (HIV-DC; *n* = 5) were stimulated with 5 μg/ml flagellin (FLG) for 24 hours. Viability, expression of characteristic DC surface markers **(A)** as well as TNF **(B)** and IL-12p70 **(C)** secretion were analyzed and compared between untreated (UN) and stimulated (FLG) conditions as well between HD-DC and HIV-DC groups 0.4 × 10^5^ FLG-treated MDDC were cultured with 4 × 10^5^ autologous lymphocytes (MDDC/lymphocytes ratio: 1/0) for 96 hours to detect IFN-γ production in CD4^+^ T lymphocytes (percentage of CD3^+^ CD4^+^ T IFN-γ^+^ cells) **(D)**, or 120 hours to measure CD4^+^ T lymphocytes proliferation (percentage of CD3^+^ CD4^+^ T CSFE^low^ cells) **(E)**. Lymphocytes alone were used as a negative control (Neg). Data are reported as mean ± standard error. Multiple *t*-test **(A)** and Two-Way ANOVA test **(B–E)** were applied to compare conditions within a group (HIV-DC or HD-DC; ^*^p < 0.05) and between groups (HIV-DC vs. HD-DC; ^§^*p* < 0.05).

FLG activates MDDC, both HIV-DC and HD-DC, as indicated by the increase of co-stimulatory molecules, cytokines release and CD4^+^ T lymphocytes activation (Figure 1).

As expected, FLG induced the up-regulation of HLA-DR (95.83 ± 0.68 % positive cells) compared to untreated cells (UN: 77.5 ± 4.4 % positive cells) and the significant increase of CD40 (FLG: 39.8 ± 3.7 %, vs. UN: 7.8 ± 2.0 % positive cells; *p* = 3 × 10^−5^) in HD-DC, but not of CD86, which appeared to be decreased in HD-DC+FLG compared to untreated cells (FLG: 62.8 ± 4.3 %, vs. UN: 91.1 ± 2.9 % positive cells; *p* = 0.001) (Figure 1A). However, this result could be due to a previously reported positive feedback mechanism and not to a negative effect of FLG (36). A significant augment of HLA-DR (FLG: 83.5 ± 6.1 %, vs. UN: 49.2 ± 9.5 % positive cells; *p* = 0.006) and CD40 (FLG: 48.7 ± 5.4 %, vs. UN: 17.4 ± 1.7 % positive cells; *p* = 0.020) was observed in HIV-DC challenged with FLG. The expression of CD86 did not change in treated or untreated cells (Figure 1A).

It is interesting to emphasize that the entity of surface markers expression did not significantly differ between HIV-DC and HD-DC in both untreated or treated conditions (Figure 1A). Even if we observed lower viability in HIV-DC compared to HD-DC, this difference did not result statistically significant (*p* > 0.999).

FLG induced the secretion of a good and similar amount of TNF in HD-DC and HIV-DC (*p* < 0.05) (Figure 1B). On the other hand, the production of IL-12 differs between HD-DC and HIV-DC (*p* = 0.029): while HD-DC produced significant level of IL-12 in response to the molecular pattern (FLG: 212.7 ± 53.1 pg/mL, vs. UN: 50.5 ± 2.9 pg/mL; *p* = 7 × 10^−4^), the induction of cytokine appeared to be less pronounced in HIV-DC (FLG: 63.9 ± 6.9 pg/mL, vs. UN: 38.2 ± 12.1 pg/mL; *p* > 0.05) (Figure 1C).

Altogether these data indicate that FLG is able to activate HIV-DC in a similar way to that seen for HD-DC.

To test whether FLG-treated MDDC are able to induce a properly adaptive immunity response, a MDDC/lymphocytes co-culture assay was performed using autologous cells as previously described (31, 32). Lymphocytes alone were used as a negative control (Neg) (Figures 1D,E).

A significant increment of IFN-γ^+^ CD4^+^ T cells was observed in healthy donors (FLG: 12.9 ± 5.5 % vs. UN: 1.3 ± 0.4 % positive cells; *p* = 0.022) as well as in HIV-infected individuals (FLG: 9.6 ± 1.8 % vs. UN: 0.1 ± 0.1 % positive cells; *p* = 0.014). Of note, the percentage of IFN-γ^+^ CD4^+^ T cells after FLG treatment was similar in healthy donor and patients (*p* > 0.999). Negative control resulted similar to untreated co-cultures (*p* > 0.05) (Figure 1D), emphasizing that the increasing percentage of positive cells is not an artifact.

Moreover, FLG-treated MDDC were able to induce a significant proliferation of CD4^+^ T lymphocytes in healthy donors (FLG: 51.0 ± 12.1 %, vs. UN: 3.7 ± 0.9 % positive cells; *p* = 0.026) and patients (FLG: 24.3 ± 2.8 %, vs. UN: 3.5 ± 0.8 % positive cells; *p* = 0.038), even if in a lesser extent in patients compared to HD (*p* = 0.004). Negative control presented a proliferation rate similar to untreated co-cultures (*p* > 0.05) (Figure 1E).

It is interesting to underline that the limited percentage of positive cells in this type of assay is in accord with previously published data for autologous MDDC and T cells co-culture both in healthy donors (32) and even in HIV-infected patients (31) treated with unspecific stimuli (mitogens). Moreover, despite its limited entity, the activation of lymphocytes is consequence of MDDC stimulation as in the absence of MDDC (negative control) mitogens cannot activate T cells (Figures 1D,E).

Taking in account the activation status of FLG-treated HIV-DC (Figures 1A–C) together with their ability to induce lymphocytes activation (Figures 1D,E), we clearly demonstrated that FLG is able to activate HIV-DC similarly to what observed for HD-DC. It is interesting to emphasize that, on the contrary, we have previously shown that HIV-DC did not properly respond to bacterial LPS (14).

### Flagellin, but Not LPS, Induces Comparable Inflammasome Activation in MDDC From HIV-Infected Patients and Healthy Donors

**Figure 2:**
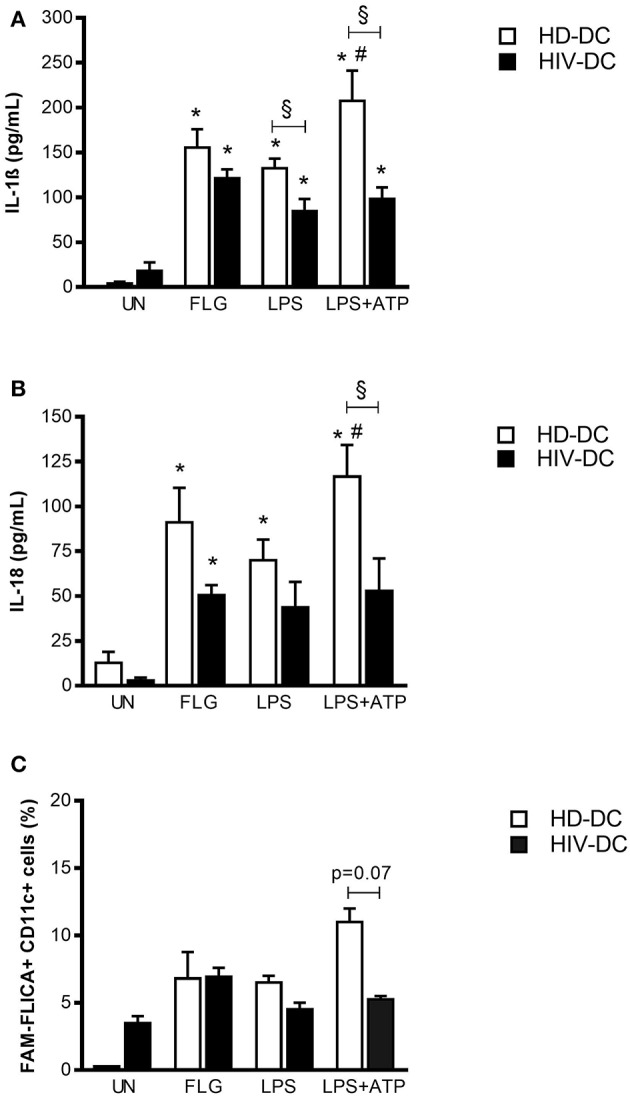
Flagellin induces better inflammasome activation in HIV-DC than LPS. 2 × 10^5^ MDDC from healthy donors (HD-DC; *n* = 20) or HIV-infected patients (HIV-DC; *n* = 20) were stimulated with 5 μg/mL flagellin (FLG) for 24 hours. 1 μg/mL LPS for 24 hours, or 1 μg/mL LPS for 24 hours plus 1 mM ATP for more 15 minutes were used as positive control for the activation of inflammasome and NLRP3 inflammasome, respectively. Culture supernatants were used to measure IL-1β **(A)** and IL-18 **(B)** concentration (pg/mL). Cells were harvested for analysis of caspase-1 activity by FAM-FLICA assay and flow cytometry. Percentage of FAM-FLICA+CD11c^+^ cells were reported for FLG-treated (FLG) and untreated (UN) MDDC **(C)**. Data are represented as mean ± standard error. Two-Way ANOVA test was applied to compare conditions within a group (HIV-DC or HD-DC; ^*^p < 0.05; LPS+ATP vs. LPS: ^#^*p* < 0.05) and between groups (HIV-DC vs. HD-DC; ^§^*p* < 0.05).

We then investigated the ability of flagellin to stimulate inflammasome in MDDC by the meaning of inflammasome cytokines production and caspase-1 activity. LPS alone or in combination with ATP was added to the assay as a positive control for inflammasome or NLRP3 inflammasome activation, respectively (26) (Figure 2). A time-course assay treating MDDC with 5 μg/mL FLG or 1 μg/mL LPS was performed to determine the best experimental time for IL-1ß detection in this model, (Supplementary File 6), and selected 24 h for all the experiments.

FLG induced a significant IL-1ß release in HD-DC (155.3 ± 20.7 pg/mL), compared to unstimulated cells (4.0 ± 1.9 pg/mL; *p* < 0.0001) and similarly to LPS (132.4 ± 9.9 pg/mL; *p* < 0.0001). The treatment with LPS+ATP resulted in a significant increase of IL-1ß release compared to LPS (*p* = 0.002), indicating a proper response of NLRP3 inflammasome in HD-DC (Figure 2A).

In HIV-DC, the entity of IL-1ß production resulted lower than in HD-DC but significantly augmented compared to resting cells (FLG: 121.3 ± 34.3 pg/mL; vs. UN: 18.3 ± 9.3 pg/mL; *p* = 2 × 10^−4^) and also to LPS-treated cells (84.4 ± 17.5 pg/mL; *p* = 0.002). However, ATP did not alter LPS-induced IL-1ß production (*p* > 0.05) (Figure 2A), confirming the previously observed dysregulation of NLRP3 inflammasome in MDDC from HIV-infected patients (14).

In a similar way, FLG also induced significantly IL-18 release in HD-DC (FLG: 91.1 ± 19.3 pg/mL, vs. UN: 12.8 ± 6.1 pg/mL; *p* = 0.018) and at lower extent in HIV-DC (FLG: 50.5 ± 5.6 pg/mL, vs. UN: 2.9 ± 1.5 pg/mL; *p* = 0.009). HD-DC better respond to LPS and LPS+ATP than HIV-DC also in term of IL-18. There were no statistical differences in the production of IL-18 between HD- and HIV-DC (Figure 2B).

Inflammasome cytokines release revealed that FLG is able to induce complex activation in MDDC from healthy as well as HIV-infected individuals. Accordingly, FLG increased caspase-1 activity in HD-DC (FLG: 6.8 ± 2.0 %, vs. UN: 0.3 ± 0.0 % positive cells) and in HIV-DC (FLG: 6.9 ± 0.7 %, vs. UN: 3.5 ± 0.5 % positive cells). HIV-DC presented a constitutively activated caspase-1, however a lower activation in response to LPS+ATP compared to HD-DC, once more emphasizing the specific dysregulation of NLRP3 pathway (Figure 2C). Even if these differences did not reach statistical significance (*p* = 0.07), we underline that caspase-1 activity accompanies above-mentioned cytokines data, and that this is the first study showing a tendency in caspase-1 activation defect in MDDC from HIV-infected individuals. Although other studies have reported statistically significant differences in caspase-1 activity in healthy donors and HIV-infected patients, those results referred to lymphoid compartment or peripheral blood mononuclear cells, while little is known in myeloid cells (37–40).

### Flagellin Induces Inflammasome Activation by Stimulated NAIP/NLRC4 and NLRP3 Receptors in HD-DC, but Not in HIV-DC

Once assessed that FLG is able to induce inflammasome activation in MDDC, we therefore tried to depict the pathways involved in complex formation and to detect any differences between HD-DC and HIV-DC.

**Figure 3:**
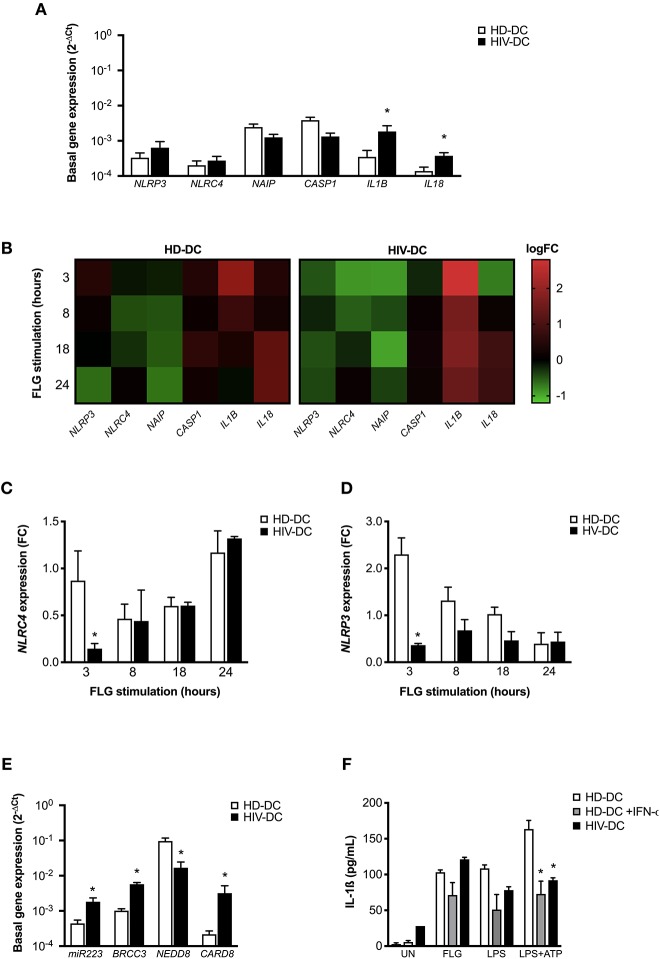
NLRC4 and NLRP3 are differentially involved in flagellin activation in HD-DC and HIV-DC. 2 × 10^5^ MDDC from healthy donors (HD-DC; *n* = 5) or HIV-infected patients (HIV-DC; *n* = 5) were stimulated with 5 μg/ml flagellin (FLG) for 3, 8, 18, and 24 hours. Cells were harvested for total RNA isolation and evaluation of relative gene expression by qPCR **(A–E)**. **(A)** Basal gene expression of *NLRP3, NAIP, NLRC4, CASP1, IL18*, and *IL1B* was expressed as 2^−Δ*Ct*^. **(B)** FLG-induced expression of *NLRP1, NLRP3, NLRC4, NAIP, CASP1, IL1B*, and *IL18* genes was calculated as 2^−ΔΔ*Ct*^ (fold-change, FC) and reported as logFC in a heat-map graph for HD-DC and HIV-DC. **(C)** FC values for *NLRC4* are compared between HD-DC and HIV-DC at all the time-points. **(D)** FC values for *NLRP3* are compared between HD-DC and HIV-DC at all the time-points. **(E)** Basal gene expression of *miR223, BRCC3, NEDD8*, and *CARD8* was expressed as 2^−Δ*Ct*^. **(F)** 2 × 10^5^ MDDC from healthy donors (HD-DC; *n* = 3) were pre-treated with 1,000 UI/mL of IFN-α-2b (Schering-Plough) for 18 h and then with 5 μg/ml flagellin (FLG) or LPS 1 μg/mL LPS for 24 hours, or 1 μg/mL LPS for 24 hours plus 1 mM ATP for more 15 minutes. IL-1ß concentration was measured in culture supernatants of HD-DC with and without IFN-α-2b pre-treatment and compared with HIV-DC (*n* = 3) stimulated with FLG. Data are represented as mean ± standard error. Multiple *t*-test was applied to compare HD- and HIV-DC in **(A,C–E)**. Kruskall-Wallis test was applied to compare HD-DC, HD-DC + IFN-α-2b, and HIV-DC sets in **(F)** (^*^p < 0.05).

Gene expression analysis revealed that HIV-DC presents a significant higher constitutive expression of *IL1B* and *IL18* compared to HD-DC. *NLRP3* also resulted increased even if not in a statistically significant way, while the level of *NAIP* and *NLRC4* was similar between MDDC (Figure 3A).

When the effect of flagellin was evaluated in genes modulation, we observed a revealed a different expression profile in HD-DC and HIV-DC at all the analyzed time-points (3, 8, 18, and 24 h) (Figure 3B).

In particular, we focused our attention on the two receptors *NLRP3* and *NLRC4*. While, as expected, FLG induces NLRC4 gene modulation in HD-DC and HIV-DC (Figure 3C), NLRP3 appeared to be defective in HIV-DC, as FLG was able to induce NLRP3 expression in HD, but not in HIV-DC (Figure 3D), according to our previously published data (14).

The dysregulation observed for NLRP3 could be due to an imbalance of inhibitor and activator signals. NLRP3 is tightly regulated by endogenous proteins CARD8 (41), BRCC3 (42) and NEDD8 (43), and by miR-223 (44). Interestingly, the basal expression of *CARD8* and *BRCC3*, as well as of miR-223, resulted significantly augmented in HIV-DC compared to HD-DC (Figure 3E), suggesting a possible cause of low responsiveness of NLRP3. Moreover, taking in account that IFN-I also contributes to the negative regulation of NLRP3 (29, 30) and that HIV-infected patients are known to present high level of circulating IFN-I (45–47), we shown that the treatment of HD-DC with IFN-a significantly reduced IL-1ß release specifically in LPS+ATP treated cells up to cytokine level observed in HIV-DC (Figure 3F), emphasizing the inhibitory role of IFN-I on NLRP3 and suggesting that this could be another cause of a specific NLRP3 defect in HIV-DC.

According to our initial hypothesis, flagellin appears to be able to by-pass this defect of NLRP3 in HIV-DC, and at the same time our results have shown the involvement of both NLRC4 and NLRP3 receptors in HD-DC response to flagellin.

**Figure 4:**
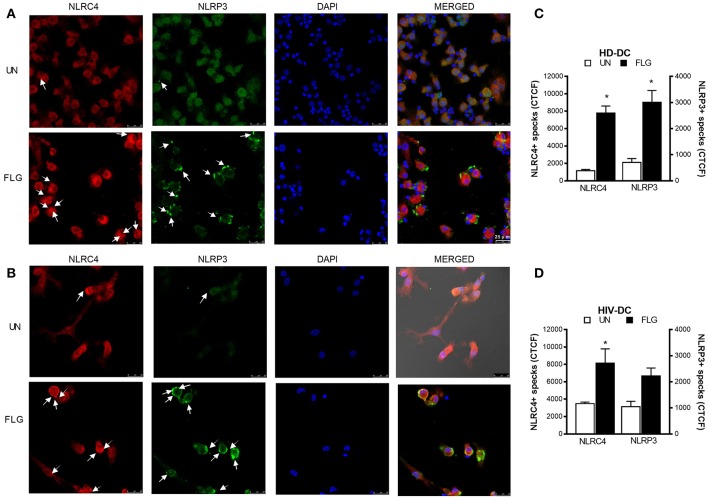
Flagellin induces NLRC4+ and NLRP3+ specks formation in MDDC. 2 × 10^5^ MDDC from healthy donors (HD-DC; *n* = 3) or HIV-infected patients (HIV-DC; *n* = 3) were cultured in 16-wells chamber slides and stimulated with 5 μg/mL FLG or 1 μg/mL LPS for 24 hours at 37°C 5% CO_2_ with or without 1 mM ATP for more 15 minutes. Mouse anti-human NLRP3 and rabbit anti-human NLRC4 antibodies and fluorescent secondary antibodies (Alexa 488-conjugated goat-anti-mouse IgG1; Alexa 647-conjugated goat-anti-rabbit IgG1) were used to label NLRP3+ and NLRC4+ specks, respectively. DAPI was used to counterstain nuclei. Images acquisition was performed using a DMi8 confocal laser scanning microscope. A representative experiment (magnification: 63x) was reported for HD-DC **(A)** and HIV-DC **(B)**. Arrows indicated NLRC4+ or NLRP3+ specks. **(C,D)** CTCF index for NLRP3+ and NLRC4+ specks in untreated (UN) and FLG-treated (FLG) MDDC. Data are represented as mean ± standard error. Mann-Whitney test was applied to compare UN and FLG conditions in **(C,D)** (^*^p < 0.05).

This hypothesis of a “two-receptors” mechanism is supported also by inflammasome “specks” detection through immunofluorescence staining of NLRP3 and NLRC4 (Figure 4). NLRC4+ specks were evidenced in confocal imagines of FLG-treated HD-DC (Figure 4A) and HIV-DC (Figure 4B). NLRP3+ specks resulted more in FLG-treated HD-DC (Figure 4A) than in HIV-DC (Figure 4B). In general NLRC4 and NLRP3 staining localized in the same cells. By the use of CTCF index, we showed that FLG significantly induced both NLRC4+ and NLRP3+ specks in HD-DC (FLG vs. UN: *p* < 0.0001) (Figure 4C). On the other hands, in HIV-DC FLG induced preferentially NLRC4+ specks (CTCF FLG vs. UN: *p* = 0.0001) and at lesser extent NLRP3+ specks (*p* > 0.05) (Figure 4D). These findings demonstrated that in healthy donor cells, FLG not only activates inflammasome through the expected NAIP/NLRC4 pathway but also through the NLRP3 one.

**Figure 5:**
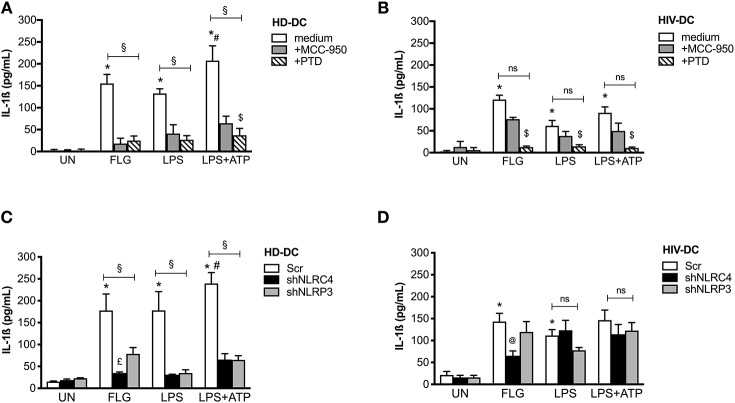
Flagellin significantly induces NLRC4 but not NLRP3 in HIV-DC. 2 × 10^5^ MDDC from healthy donors (HD-DC; *n* = 5) **(A)** and HIV-infected patients (HIV-DC; *n* = 5) **(B)** were pretreated with 10 μM MCC-950 or 10 μM parthenolide (PTD), and then stimulated with 5 μg/ml FLG for 24 hours or 1 μg/mL LPS for 24 hours with or without 1 mM ATP for more 15 minutes. IL-1ß concentration was measured in culture supernatants. 2 × 10^5^ MDDC from healthy donors (HD-DC; *n* = 3) **(C)** and HIV-infected patients (HIV-DC; *n* = 3) **(D)** were transduced with empty vector (scramble: Scr), shNLRC4 or shNLRP3, and then stimulated with 5 μg/ml FLG for 24 hours, or 1 μg/mL LPS for 24 hours with or without 1 mM ATP for more 15 minutes. IL-1ß concentration was measured in culture supernatants. Kruskal-Wallis test with multi comparison post-test was applied (treated vs. UN: ^*^p < 0.05; LPS+ATP vs. LPS ^#^*p* < 0.05; within each group: ^§^*p* < 0.05; +MCC-950 vs. +PTD: ^$^*p* < 0.05; shNLRC4 vs. shNLRP3: £*p* < 0.05; shNLRC4 vs. Scr: @*p*<0.05).

To better investigate the involvement of NAIP/NLRC4 and NLRP3 in response to FLG in our model, we evaluated the IL-1ß production in FLG-treated MDDC previously incubated with 10 μM MCC-950, a specific NLRP3 inhibitor (27), or with 10 μM parthenolide/PTD, a large spectrum inflammasome inhibitor (28) (Figures 5A,B).

MCC-950 and PTD significantly inhibited IL-1ß production in FLG-treated HD-DC (78 and 90% of inhibition, respectively; *p* < 0.05), as well as LPS+ATP-treated cells (78 and 90% of inhibition, respectively; *p* < 0.05), and partially LPS-induced inflammasome activation (78 and 90% of inhibition, respectively; *p* < 0.05) (Figure 5A), due to the contribution of other pathway in inflammasome activation by LPS (48).

On the other side, MCC-950 was not able to significantly reduce cytokine release in HIV-DC stimulated with FLG (48% of inhibition; *p* > 0.05), LPS (48% of inhibition; *p* > 0.05) or LPS+ATP (48% of inhibition; *p* > 0.05) (Figure 5B). Accordingly, in the presence of MCC-950 a reduction not significantly of NLRP3+ specks was observed in HIV-DC (Supplementary File 7). PTD similarly inhibited IL-1β production in both FLG-treated HIV-DC (84% of inhibition *p* = 0.002), LPS or LPS+ATP-treated HIV-DC (84% of inhibition; *p* = 0.002), as expected due to the caspase-1 and NF-kB dependency for FLG-induced response (49), as well as for LPS-mediated one (48). These data confirm the greater involvement of NLRP3 in response to FLG in healthy donors compared to HIV-infected individuals.

Then we compared the differences in IL-1ß release in FLG-treated MDDC following *NLRC4* or *NLRP3* shRNA knockdown (Figures 5C,D). In *NLRC4* shRNA-transduced MDDC, FLG induced a lower level of IL-1ß production compared to untreated cells both in HD-DC (Figure 5C) and in HIV-DC (Figure 5D). In *NLRP3* siRNA-transducted MDDC, FLG induced a lower level of IL-1ß compared to untreated cells in HD-DC (Figure 5C), however this effect was not observed in HIV-DC (Figure 5D). These data are in accord with above-reported effect of chemical NLRP3 inhibitor MCC-950 (Figures 5A,B).

Altogether these findings allow us to suggest that FLG activates the inflammasome in human MDDC through the induction of NAIP/NLRC4 and NLRP3 receptors; the contribution of NLRP3 in FLG-signaling is less pronounced in HIV-DC due to the well-known “exhausted” profile of these cells (50).

## Discussion

A number of microbial components have been proposed as alternative adjuvants to augment the immune responses of poorly immunogenic vaccines and/or of not fully immunocompetent individuals. Emerging evidence pointed out the possible use of bacterial flagellin in this context, firstly in mice [as extensively revised in Hajam et al. (17)], but also in a human clinical trial of prophylactic vaccine (23). Studies in mice show the stimulatory capacity of flagellin to induce both humoral and cellular immune responses when implied together with pathogen' antigens as adjuvant (51, 52) or in cancer immunotherapy (53).

Taking in account that, even in ART treatment, HIV-infected individuals continue to experience immune dysfunction, leading, among others side effects, to a deficient vaccine response (9), the necessity of new vaccine strategy in this population appear to be urgent.

Using the *in vitro* model of peripheral blood monocytes-derived-dendritic cells developed for HIV-infected patient's immunotherapy by Lu and collaborators (54), we demonstrated that flagellin is able to activate MDDC from HIV-infected patients as well as from healthy donors. Previous studies have evidenced the ability of flagellin to activate primary human monocytes, or human pro-monocytic cell line U38 (55), however here we demonstrated that flagellin is able to activate human MDDC considering both the MDDC profile as well as the MDDC-mediated lymphocytes activation (Figure 1), with the exception of IL-12 production which results lower in HIV-DC compared to HD-DC, but in accord with previously published data (56).

Flagellin is sensed by two main innate immune receptors, TLR5 (18) and NAIP/NLRC4 (57–59). While it has been shown that flagellin induces NAIP/NLRC4 inflammasome activation in primary human macrophages and monocytes (49, 60, 61), little is known about its role in human DC. Despite it has been previously reported that flagellin stimulates IL-1β production in human MDDC (62), any evidence about NLRC4 or NLRP3 pathway in IL-1ß induction have been shown nor hypothesized. Besides the description of these two receptors within inflammasome activation by flagellin, our study also demonstrated for the first time the different contribution of the two receptors in HIV-DC response to flagellin.

As expected, flagellin induces the inflammasome activation through NAIP/NLRC4 both in HD-DC as well as HIV-DC (Figure 2), suggesting that this pathway is still effective in HIV-infected patients, contrary to what seen for NLRP3 (14). Actually, HIV-DC presents a basal increased expression of NLRP3 inhibitory molecules, namely CARD8 (41), BRCC3 (42) and miR-223 (44), compatible with specific inhibition of this receptor (Figure 3E).

While the low rate of NLRP3 response in HIV-DC has been attributed to the chronic inflammation of HIV-infected patients, consequent of HIV-1 persistence, endogenous viruses reactivation (i.e., herpes simplex virus), immune system exhaustion, increased intestinal permeability and microbial translocation (mainly LPS) and antiretroviral drugs cytotoxicity (15, 63) and taking in account that flagellate bacteria also could be present in gut microbiota, it remains obscure why the NAIP/NLRC4 pathway continues “ready-to-go.” The administration of continues doses of LPS was found to be tolerogenic in mice (64), however any data are available about flagellin. We speculated that the increased IFN-a production in HIV-infected individuals (even if at lower levels in ART-treated patients) could be a possible cause of specific NLRP3 inhibition due to the known effect of the anti-viral mediator as NLRP3 negative regulators (30). It is important to underline that our cohort of ART-treated HIV-infected individuals presented a mean value of plasma IFN-a of about 50 pg/mL, 5-fold more than healthy individuals (about 10 pg/mL) (data not shown). Moreover, when HD-DC were treated with IFN-a their NLRP3 response is diminished similarly to what observed in HIV-DC (Figure 3F), supporting our hypothesis. Another possible explication concerns the higher constitutive recruitment of adaptor molecule ASC (Apoptosis speck like with a CARD) in inflammasome complex in HIV-infected patients compared to healthy controls as recently observed by Ahmad and colleagues (65) in PBMC. In this case, inflammasome receptors, which need ASC to mount the complex (i.e., NLRP3), could be disadvantaged in respect to sensors that directly recruit caspase-1, such as NLRC4.

Beyond the main purpose of this article, and for the first time to our knowledge, we demonstrated that NLRP3 also contribute to flagellin response in human MDDC, as revealed by NLRP3+ specks formation in FLG-treated cells (Figure 4) and by specific (chemical and genetic) inhibition of this receptor (Figure 5). Our findings are in lines with previous reports about NLRC4 and NLRP3 co-localization into a unique complex in HEK293 cells (66) and in mice bone marrow-derived macrophages during *Salmonella* infection (67).

Therefore, flagellin induces the two pathways in HD-DC, whereas it preferentially activates NLRC4 pathway in HIV-DC due to the defect in NLRP3 in these cells. Despite the immunofluorescence visualization of both NLRP3+ and NLRC4+ specks, our data are not sufficient to determine whether the two receptors are truly co-localized in the same inflammasome complex, and further investigations will be needed to finally prove it.

In conclusion, our data support the use of flagellin in the design of future vaccines effective also in immunocompromised individuals, such as HIV-infected patients. According to our findings, flagellin activates human MDDC from healthy and HIV-infected individuals through the NAIP/NLRC4 inflammasome, with the participation of NLRP3 at least in healthy donors cells. Flagellin was able to by-pass the NLRP3 defect in HIV-DC, contributing to inflammasome activation and consequently full MDDC maturation in HIV-infected patients.

## Ethics Statement

All research involving human participants was approved by the Institutional Ethics Committees of Oswaldo Cruz Hospital, Hospital das Clinicas/Faculty of Medicine of the University of São Paulo (FMUSP) and of the Institute of Biomedical Science (ICB/USP). Written informed consent was obtained from all participants, and clinical investigations were conducted according to the principles expressed in the Declaration of Helsinki.

## Author Contributions

EdR, BdA, and AP: conceived and designed experiments. EdR: performed MDDC experiments. VL: collection of samples. DdL: performed the immunofluorescence experiments. FF and JdS: ELISA. EdR, DdL, and AP: statistical analysis. EdR, DdL, VL, FF, and AP: discussion of results. EdR and AP: wrote the article. BdA: designed and discussed silencing experiments.

### Conflict of Interest Statement

The authors declare that the research was conducted in the absence of any commercial or financial relationships that could be construed as a potential conflict of interest.

## References

[1] Steinman RM. The dendritic cell system and its role in immunogenicity. Annu Rev Immunol. (1991) 9:271–96. 10.1146/annurev.iy.09.040191.001415 1910679

[2] Banchereau J Steinman RM. Dendritic cells and the control of immunity. Nature. (1998) 392:245–52. 10.1038/32588 9521319

[3] Wu L Dakic A. Development of dendritic cell system. Cell Mol Immunol. (2004) 1:112–8. 16212897

[4] Steinman RM. Dendritic cells: understanding immunogenicity. Eur J Immunol. (2007) (37Suppl. 1):S53–60. 10.1002/eji.200737400 17972346

[5] Maisonneuve C Bertholet S Philpott DJ De Gregorio E. Unleashing the potential of NOD- and Toll-like agonists as vaccine adjuvants. Proc Natl Acad Sci USA. (2014) 111:12294. 10.1073/pnas.1400478111 25136133 PMC4151741

[6] Li H Willingham SB Ting JPY Re F. Cutting edge: inflammasome activation by alum and alum's adjuvant effect are mediated by NLRP3. J Immunol. (2008) 181:17. 10.4049/jimmunol.181.1.17 18566365 PMC2587213

[7] Surendran N Nicolosi T Pichichero M. Infants with low vaccine antibody responses have altered innate cytokine response. Vaccine. (2016) 34:5700–3. 10.1016/j.vaccine.2016.09.050 27745950 PMC5261827

[8] Van Deventer HW Burgents JE Wu QP Woodford RMT Brickey WJ Allen IC. The inflammasome component Nlrp3 impairs antitumor vaccine by enhancing the accumulation of tumor-associated myeloid-derived suppressor cells. Cancer Res. (2010) 70:10161–9. 10.1158/0008-5472.CAN-10-1921 21159638 PMC3059219

[9] Geretti AM Doyle T. Immunization for HIV-positive individuals. Curr Opin Infect Dis. (2010) 23:32–8. 10.1097/QCO.0b013e328334fec4 19949327

[10] Donaghy H Gazzard B Gotch F Patterson S. Dysfunction and infection of freshly isolated blood myeloid and plasmacytoid dendritic cells in patients infected with HIV-1. Blood. (2003) 101:4505–11. 10.1182/blood-2002-10-3189 12576311

[11] Sabado RL O'brien M Subedi A Qin L Hu N Taylor E. Evidence of dysregulation of dendritic cells in primary HIV infection. Blood. (2010) 116:3839–52. 10.1182/blood-2010-03-273763 20693428 PMC2981539

[12] Cardone M Ikeda KN Varano B Gessani S Conti L. HIV-1-induced impairment of dendritic cell cross talk with γδ T lymphocytes. J Virol. (2015) 89:4798–808. 10.1128/JVI.03681-14 25673717 PMC4403483

[13] Liu J Ostrowski M. Development of targeted adjuvants for HIV-1 vaccines. AIDS Res Ther. (2017) 14:43. 10.1186/s12981-017-0165-8 28893282 PMC5594534

[14] Pontillo A Silva LT Oshiro TM Finazzo C Crovella S Duarte AJ HIV-1 induces NALP3-inflammasome expression and interleukin-1beta secretion in dendritic cells from healthy individuals but not from HIV-positive patients. AIDS. (2012) 26:11–8. 10.1097/QAD.0b013e32834d697f 21971358

[15] Deeks SG Tracy R Douek DC. Systemic effects of inflammation on health during chronic HIV infection. Immunity. (2013) 39:633–45. 10.1016/j.immuni.2013.10.001 24138880 PMC4012895

[16] Kool M Pétrilli V De Smedt T Rolaz A Hammad H Van Nimwegen M. Cutting edge: alum adjuvant stimulates inflammatory dendritic cells through activation of the NALP3 inflammasome. J Immunol. (2008) 181:3755–9. 10.4049/jimmunol.181.6.3755 18768827

[17] Hajam IA Dar PA Shahnawaz I Jaume JC Lee JH. Bacterial flagellin-a potent immunomodulatory agent. Exp Mol Med. (2017) 49:e373. 10.1038/emm.2017.172 28860663 PMC5628280

[18] Hayashi F Smith KD Ozinsky A Hawn TR Yi EC Goodlett DR. The innate immune response to bacterial flagellin is mediated by Toll-like receptor 5. Nature. (2001) 410:1099–103. 10.1038/35074106 11323673

[19] Yoon SI Kurnasov O Natarajan V Hong M Gudkov AV Osterman AL. Structural basis of TLR5-flagellin recognition and signaling. Science. (2012) 335:859–64. 10.1126/science.1215584 22344444 PMC3406927

[20] Diebolder CA Halff EF Koster AJ Huizinga EG Koning RI. Cryoelectron tomography of the NAIP5/NLRC4 inflammasome: implications for NLR activation. Structure. (2015) 23:2349–57. 10.1016/j.str.2015.10.001 26585513

[21] Hu Z Zhou Q Zhang C Fan S Cheng W Zhao Y. Structural and biochemical basis for induced self-propagation of NLRC4. Science. (2015) 350:399–404. 10.1126/science.aac5489 26449475

[22] Zhang L Chen S Ruan J Wu J Tong AB Yin Q. Cryo-EM structure of the activated NAIP2-NLRC4 inflammasome reveals nucleated polymerization. Science. (2015) 350:404–9. 10.1126/science.aac5789 26449474 PMC4640189

[23] Treanor JJ Taylor DN Tussey L Hay C Nolan C Fitzgerald T. Safety and immunogenicity of a recombinant hemagglutinin influenza-flagellin fusion vaccine (VAX125) in healthy young adults. Vaccine. (2010) 28:8268–74. 10.1016/j.vaccine.2010.10.009 20969925

[24] Turley CB Rupp RE Johnson C Taylor DN Wolfson J Tussey L. Safety and immunogenicity of a recombinant M2e-flagellin influenza vaccine (STF2.4xM2e) in healthy adults. Vaccine. (2011). 29:5145–52. 10.1016/j.vaccine.2011.05.041 21624416

[25] Sallusto F Palermo B Lenig D Miettinen M Matikainen S Julkunen I. Distinct patterns and kinetics of chemokine production regulate dendritic cell function. Eur J Immunol. (1999) 29:1617–25. 10.1002/(SICI)1521-4141(199905)29:05<1617::AID-IMMU1617>3.0.CO;2-3 10359116

[26] Gattorno M Tassi S Carta S Delfino L Ferlito F Pelagatti MA. Pattern of interleukin-1beta secretion in response to lipopolysaccharide and ATP before and after interleukin-1 blockade in patients with CIAS1 mutations. Arthritis Rheum. (2007) 56:3138–48. 10.1002/art.22842 17763411

[27] Coll RC Robertson AA Chae JJ Higgins SC Munoz-Planillo R Inserra MC. A small-molecule inhibitor of the NLRP3 inflammasome for the treatment of inflammatory diseases. Nat Med. (2015) 21:248–55. 10.1038/nm.3806 25686105 PMC4392179

[28] Juliana C Fernandes-Alnemri T Wu J Datta P Solorzano L Yu JW. Anti-inflammatory compounds parthenolide and Bay 11-7082 are direct inhibitors of the inflammasome. J Biol Chem. (2010) 285:9792–802. 10.1074/jbc.M109.082305 20093358 PMC2843228

[29] Guarda G Braun M Staehli F Tardivel A Mattmann C Forster I. Type I interferon inhibits interleukin-1 production and inflammasome activation. Immunity. (2011) 34:213–23. 10.1016/j.immuni.2011.02.006 21349431

[30] Simmons DP Wearsch PA Canaday DH Meyerson HJ Liu YC Wang Y. Type I IFN drives a distinctive dendritic cell maturation phenotype that allows continued class II MHC synthesis and antigen processing. J Immunol. (2012) 188:3116–26. 10.4049/jimmunol.1101313 22371391 PMC3311734

[31] Da Silva LT Da Silva WC De Almeida A Da Silva Reis D Santillo BT Rigato PO. Characterization of monocyte-derived dendritic cells used in immunotherapy for HIV-1-infected individuals. Immunotherapy. (2018) 10:871–85. 10.2217/imt-2017-0165 30073900

[32] Shinde P Fernandes S Melinkeri S Kale V Limaye L. Compromised functionality of monocyte-derived dendritic cells in multiple myeloma patients may limit their use in cancer immunotherapy. Sci Rep. (2018) 8:5705. 10.1038/s41598-018-23943-w 29632307 PMC5890285

[33] Schmittgen TD Livak KJ. Analyzing real-time PCR data by the comparative C(T) method. Nat Protoc. (2008) 3:1101–8. 10.1038/nprot.2008.73 18546601

[34] Stutz A Horvath GL Monks BG Latz E. ASC speck formation as a readout for inflammasome activation. Methods Mol Biol. (2013) 1040:91–101. 10.1007/978-1-62703-523-1_8 23852599

[35] Mccloy RA Rogers S Caldon CE Lorca T Castro A Burgess A. Partial inhibition of Cdk1 in G 2 phase overrides the SAC and decouples mitotic events. Cell Cycle. (2014) 13:1400–12. 10.4161/cc.28401 24626186 PMC4050138

[36] Baravalle G Park H Mcsweeney M Ohmura-Hoshino M Matsuki Y Ishido S. Ubiquitination of CD86 is a key mechanism in regulating antigen presentation by dendritic cells. J Immunol. (2011). 187:2966–73. 10.4049/jimmunol.1101643 21849678 PMC4496154

[37] Doitsh G Galloway NLK Geng X Yang Z Monroe KM Zepeda O. Cell death by pyroptosis drives CD4 T-cell depletion in HIV-1 infection. Nature. (2013) 505:509. 10.1038/nature12940 24356306 PMC4047036

[38] Walsh JG Reinke SN Mamik MK Mckenzie BA Maingat F Branton WG. Rapid inflammasome activation in microglia contributes to brain disease in HIV/AIDS. Retrovirology. (2014) 11:35. 10.1186/1742-4690-11-35 24886384 PMC4038111

[39] Bandera A Masetti M Fabbiani M Biasin M Muscatello A Squillace N. The NLRP3 inflammasome is upregulated in HIV-infected antiretroviral therapy-treated individuals with defective immune recovery. Front Immunol. (2018) 9:214. 10.3389/fimmu.2018.00214 29483915 PMC5816335

[40] Feria MG Taborda NA. HIV replication is associated to inflammasomes activation, IL-1beta, IL-18 and caspase-1 expression in GALT and peripheral Blood. PLoS ONE. (2018) 13:e0192845. 10.1371/journal.pone.0192845 29672590 PMC5909617

[41] Ito S Hara Y Kubota T. CARD8 is a negative regulator for NLRP3 inflammasome, but mutant NLRP3 in cryopyrin-associated periodic syndromes escapes the restriction. Arthritis Res Ther. (2014) 16:R52. 10.1186/ar4483 24517500 PMC4060228

[42] Py BF Kim MS Vakifahmetoglu-Norberg H Yuan J. Deubiquitination of NLRP3 by BRCC3 critically regulates inflammasome activity. Mol Cell. (2013) 49:331–8. 10.1016/j.molcel.2012.11.009 23246432

[43] Segovia JA Tsai S-Y Chang T-H Shil NK Weintraub ST Short JD. Nedd8 regulates inflammasome-dependent caspase-1 activation. Mol Cell Biol. (2015) 35:582–97. 10.1128/MCB.00775-14 25452302 PMC4285429

[44] Yang Z Zhong L Xian R Yuan B. MicroRNA-223 regulates inflammation and brain injury via feedback to NLRP3 inflammasome after intracerebral hemorrhage. Mol Immunol. (2015) 65:267–76. 10.1016/j.molimm.2014.12.018 25710917

[45] Von Sydow M Sonnerborg A Gaines H Strannegard O. Interferon-alpha and tumor necrosis factor-alpha in serum of patients in various stages of HIV-1 infection. AIDS Res Hum Retrovirus. (1991) 7:375–80. 10.1089/aid.1991.7.375 1906289

[46] Khatissian E Tovey MG Cumont MC Monceaux V Lebon P Montagnier L. The relationship between the interferon alpha response and viral burden in primary SIV infection. AIDS Res Hum Retrovirus. (1996) 12:1273–8. 10.1089/aid.1996.12.1273 8870849

[47] Stylianou E Aukrust P Bendtzen K Muller F Froland SS. Interferons and interferon (IFN)-inducible protein 10 during highly active anti-retroviral therapy (HAART)-possible immunosuppressive role of IFN-alpha in HIV infection. Clin Exp Immunol. (2000) 119:479–85. 10.1046/j.1365-2249.2000.01144.x 10691920 PMC1905596

[48] Kayagaki N Wong MT Stowe IB Ramani SR Gonzalez LC Akashi-Takamura S. Noncanonical inflammasome activation by intracellular LPS independent of TLR4. Science. (2013) 341:1246–9. 10.1126/science.1240248 23887873

[49] Kortmann J Brubaker SW Monack DM. Cutting edge: inflammasome activation in primary human macrophages is dependent on flagellin. J Immunol. (2015) 195:815–9. 10.4049/jimmunol.1403100 26109648 PMC4505955

[50] Blanchet FP Moris A Nikolic DS Lehmann M Cardinaud S Stalder R. Human immunodeficiency virus-1 inhibition of immunoamphisomes in dendritic cells impairs early innate and adaptive immune responses. Immunity. (2010) 32:654–69. 10.1016/j.immuni.2010.04.011 20451412 PMC2929482

[51] Mcsorley SJ Ehst BD Yu Y Gewirtz AT. Bacterial flagellin is an effective adjuvant for CD4+ T cells *in vivo*. J Immunol. (2002) 169:3914–9. 10.4049/jimmunol.169.7.3914 12244190

[52] Didierlaurent A Ferrero I Otten LA Dubois B Reinhardt M Carlsen H. Flagellin promotes myeloid differentiation factor 88-dependent development of Th2-type response. J Immunol. (2004) 172:6922–30. 10.4049/jimmunol.172.11.6922 15153511

[53] Zheng JH Nguyen VH Jiang SN Park SH Tan W Hong SH. Two-step enhanced cancer immunotherapy with engineered Salmonella typhimurium secreting heterologous flagellin. Sci Transl Med. (2017) 9:9537. 10.1126/scitranslmed.aak9537 28179508

[54] Lu W Arraes LC Ferreira WT Andrieu JM. Therapeutic dendritic-cell vaccine for chronic HIV-1 infection. Nat Med. (2004) 10:1359–65. 10.1038/nm1147 15568033

[55] Ciacci-Woolwine F Blomfield IC Richardson SH Mizel SB. Salmonella flagellin induces tumor necrosis factor alpha in a human promonocytic cell line. Infect Immun. (1998) 66:1127–34. 9488405 10.1128/iai.66.3.1127-1134.1998 PMC108025

[56] Buisson S Benlahrech A Gazzard B Gotch F Kelleher P Patterson S. Monocyte-derived dendritic cells from HIV type 1-infected individuals show reduced ability to stimulate T cells and have altered production of interleukin (IL)-12 and IL-10. J Infect Dis. (2009) 199:1862–71. 10.1086/599122 19419334

[57] Franchi L Amer A Body-Malapel M Kanneganti TD Ozoren N Jagirdar R. Cytosolic flagellin requires Ipaf for activation of caspase-1 and interleukin 1beta in salmonella-infected macrophages. Nat Immunol. (2006) 7:576–82. 10.1038/ni1346 16648852

[58] Miao EA Alpuche-Aranda CM Dors M Clark AE Bader MW Miller SI. Cytoplasmic flagellin activates caspase-1 and secretion of interleukin 1beta via Ipaf. Nat Immunol. (2006) 7:569–75. 10.1038/ni1344 16648853

[59] Kofoed EM Vance RE. Innate immune recognition of bacterial ligands by NAIPs determines inflammasome specificity. Nature. (2011) 477:592–5. 10.1038/nature10394 21874021 PMC3184209

[60] Canna SW De Jesus AA Gouni S Brooks SR Marrero B Liu Y. An activating NLRC4 inflammasome mutation causes autoinflammation with recurrent macrophage activation syndrome. Nat Genet. (2014) 46:1140–6. 10.1038/ng.3089 25217959 PMC4177369

[61] Reyes Ruiz VM Ramirez J Naseer N Palacio NM Siddarthan IJ Yan BM. Broad detection of bacterial type III secretion system and flagellin proteins by the human NAIP/NLRC4 inflammasome. Proc Natl Acad Sci USA. (2017) 114:13242. 10.1073/pnas.1710433114 29180436 PMC5740664

[62] Means TK Hayashi F Smith KD Aderem A Luster AD. The toll-like receptor 5 stimulus bacterial flagellin induces maturation and chemokine production in human dendritic cells. J Immunol. (2003) 170:5165. 10.4049/jimmunol.170.10.5165 12734364

[63] Maartens G Celum C Lewin SR. HIV infection: epidemiology, pathogenesis, treatment, and prevention. Lancet. (2014) 384:258–71. 10.1016/S0140-6736(14)60164-1 24907868

[64] Geisel J Kahl F Müller M Wagner H Kirschning CJ Autenrieth IB. IL-6 and maturation govern TLR2 and TLR4 induced TLR agonist tolerance and cross-tolerance in dendritic cells. J Immunol. (2007) 179:5811. 10.4049/jimmunol.179.9.5811 17947654

[65] Ahmad F Mishra N Ahrenstorf G Franklin BS Latz E Schmidt RE. Evidence of inflammasome activation and formation of monocyte-derived ASC specks in HIV-1 positive patients. AIDS. (2018) 32:299–307. 10.1097/QAD.0000000000001693 29135573

[66] Man SM Hopkins LJ Nugent E Cox S Glück IM Tourlomousis P. Inflammasome activation causes dual recruitment of NLRC4 and NLRP3 to the same macromolecular complex. Proc Natl Acad Sci USA. (2014) 111:7403–8. 10.1073/pnas.1402911111 24803432 PMC4034195

[67] Qu Y Misaghi S Newton K Maltzman A Izrael-Tomasevic A Arnott D. NLRP3 recruitment by NLRC4 during *Salmonella* infection. J Exp Med. (2016) 213:877–85. 10.1084/jem.20132234 27139490 PMC4886354

