# Supplementary material for: Flagellin/NLRC4 Pathway Rescues NLRP3-Inflammasome Defect in Dendritic Cells From HIV-Infected Patients: Perspective for New Adjuvant in Immunocompromised Individuals
Source: Front Immunol. 2019 Jun 11;10:1291. doi: 10.3389/fimmu.2019.01291 (PMC6579915; doi:10.3389/fimmu.2019.01291)
Supplement: Supplementary file 1 [file Presentation_1.pptx]

## Slide 1
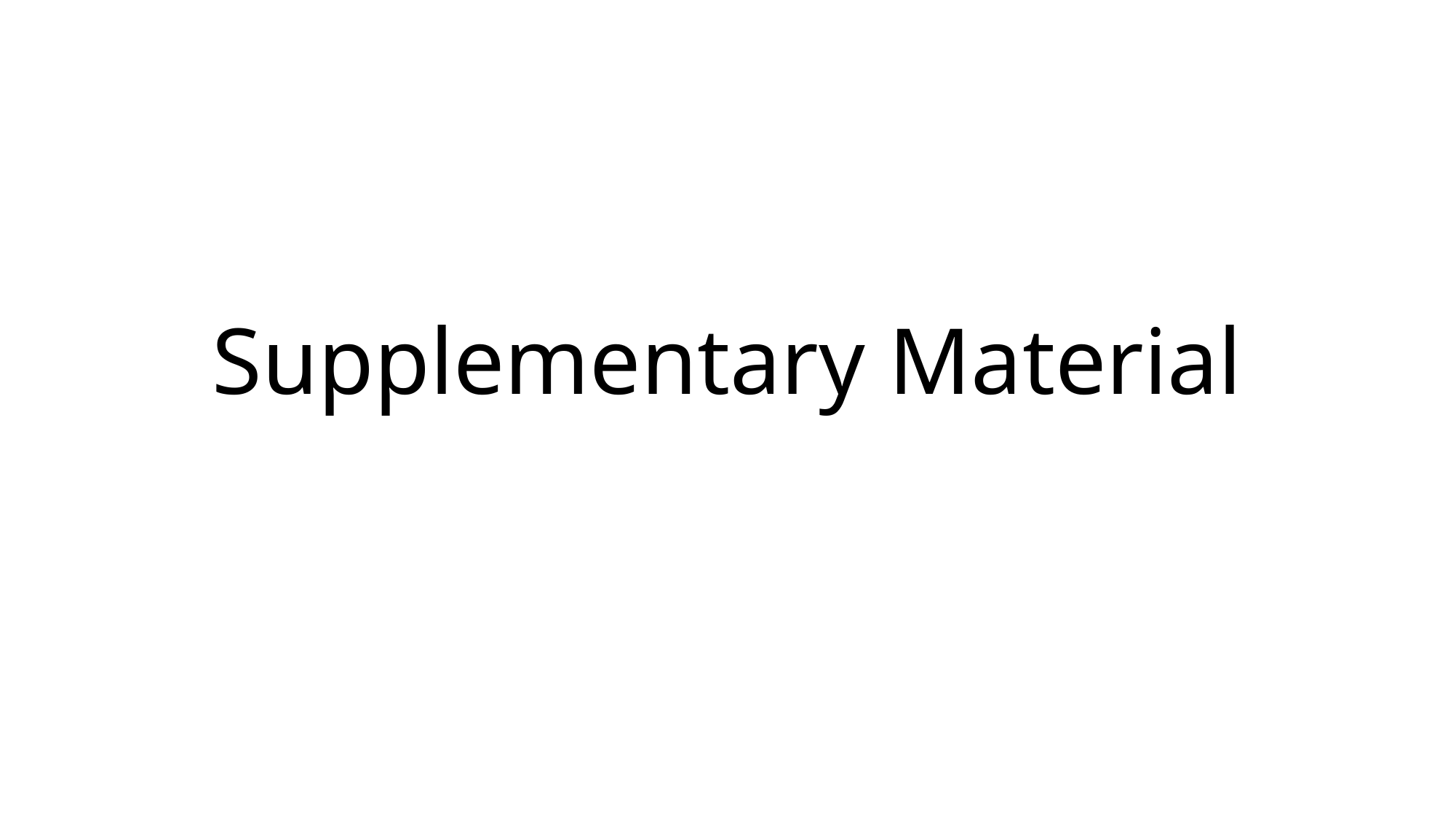

# Supplementary Material

## Slide 2
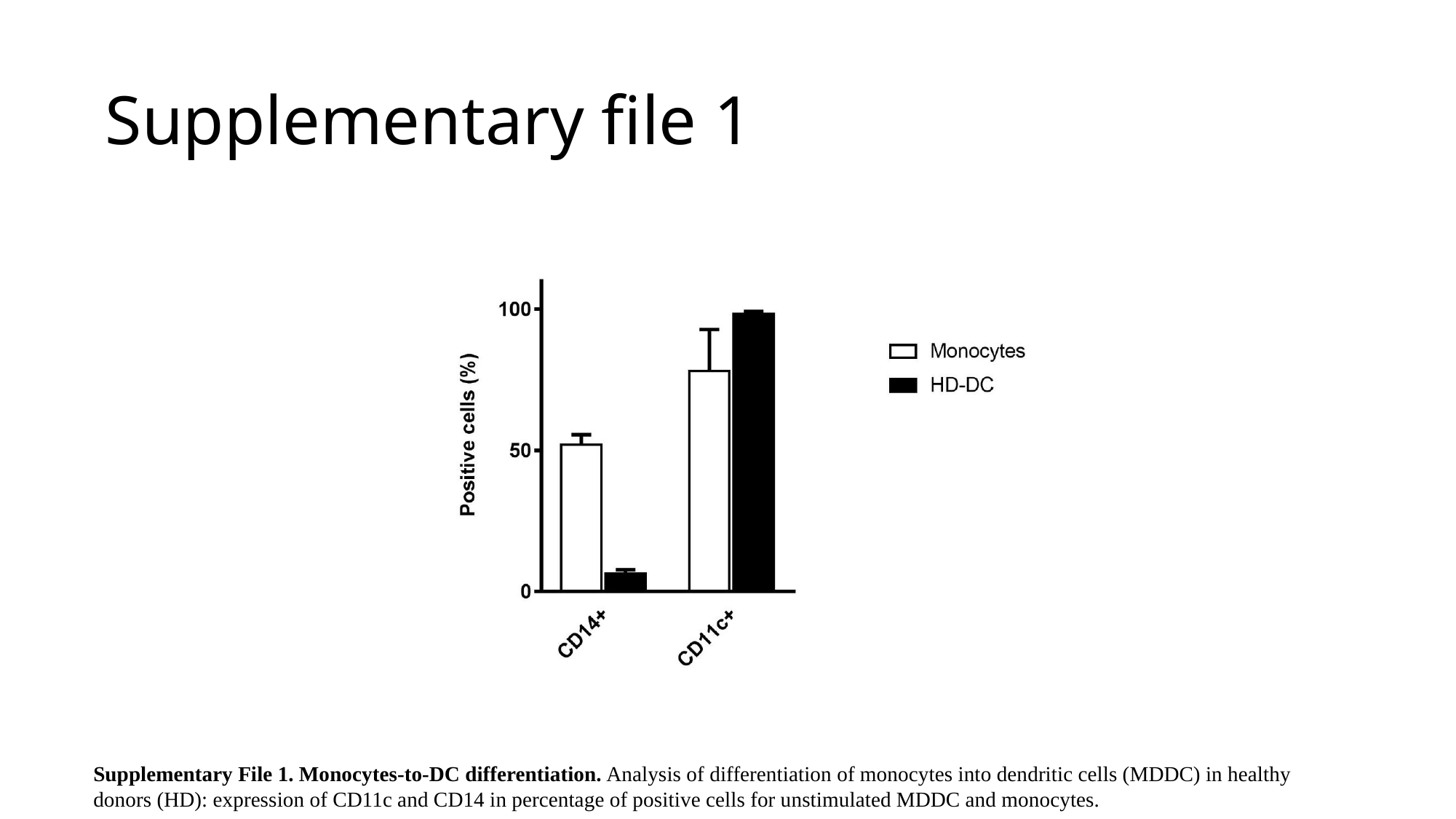

# Supplementary file 1
Supplementary File 1. Monocytes-to-DC differentiation. Analysis of differentiation of monocytes into dendritic cells (MDDC) in healthy donors (HD): expression of CD11c and CD14 in percentage of positive cells for unstimulated MDDC and monocytes.

## Slide 3
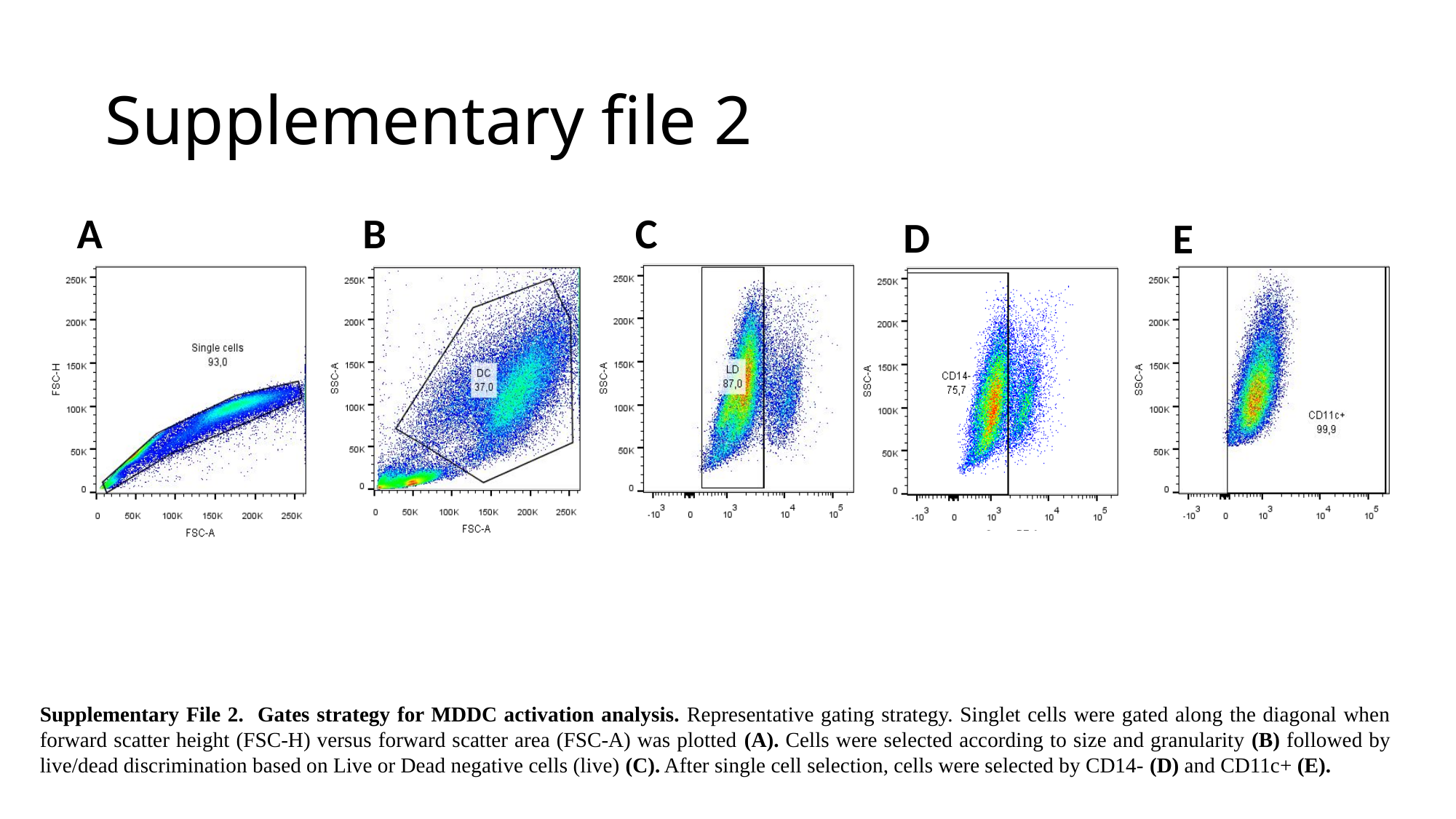

# Supplementary file 2
A
B
C
D
E
Supplementary File 2. Gates strategy for MDDC activation analysis. Representative gating strategy. Singlet cells were gated along the diagonal when forward scatter height (FSC-H) versus forward scatter area (FSC-A) was plotted (A). Cells were selected according to size and granularity (B) followed by live/dead discrimination based on Live or Dead negative cells (live) (C). After single cell selection, cells were selected by CD14- (D) and CD11c+ (E).

## Slide 4
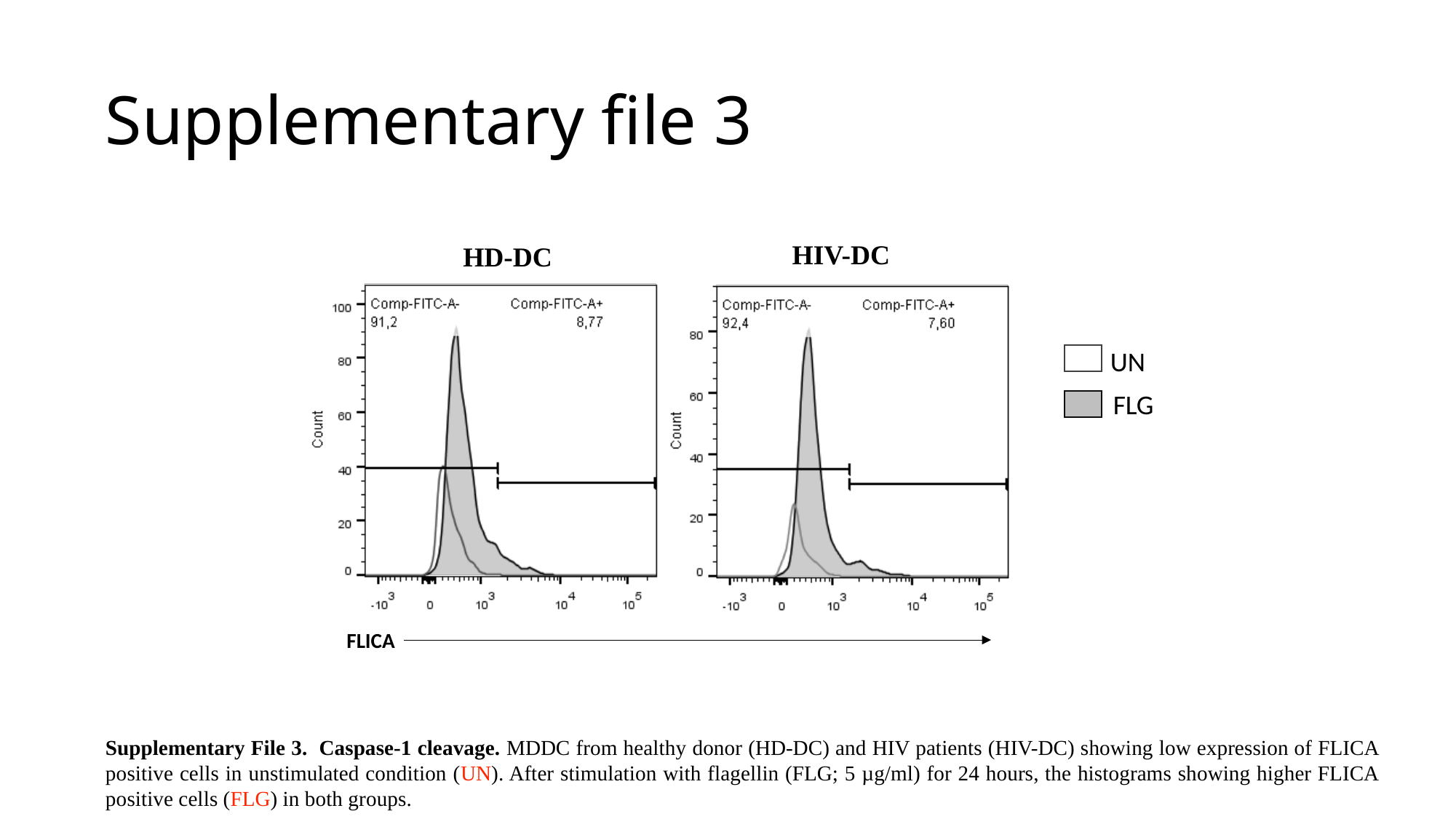

# Supplementary file 3
HIV-DC
HD-DC
UN
FLG
FLICA
Supplementary File 3. Caspase-1 cleavage. MDDC from healthy donor (HD-DC) and HIV patients (HIV-DC) showing low expression of FLICA positive cells in unstimulated condition (UN). After stimulation with flagellin (FLG; 5 µg/ml) for 24 hours, the histograms showing higher FLICA positive cells (FLG) in both groups.

## Slide 5
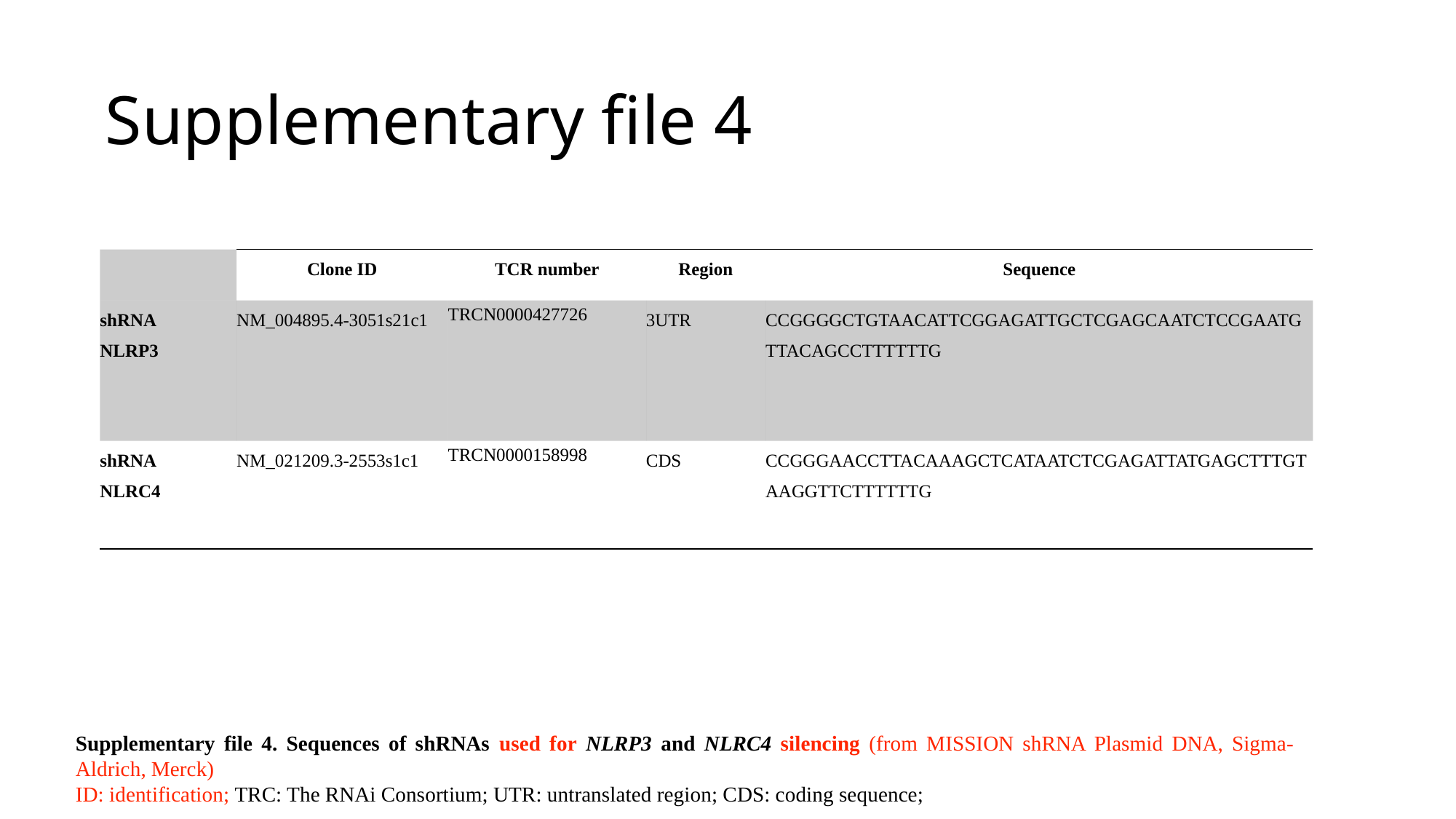

# Supplementary file 4
| | Clone ID | TCR number | Region | Sequence |
| --- | --- | --- | --- | --- |
| shRNA NLRP3 | NM\_004895.4-3051s21c1 | TRCN0000427726 | 3UTR | CCGGGGCTGTAACATTCGGAGATTGCTCGAGCAATCTCCGAATGTTACAGCCTTTTTTG |
| shRNA NLRC4 | NM\_021209.3-2553s1c1 | TRCN0000158998 | CDS | CCGGGAACCTTACAAAGCTCATAATCTCGAGATTATGAGCTTTGTAAGGTTCTTTTTTG |
Supplementary file 4. Sequences of shRNAs used for NLRP3 and NLRC4 silencing (from MISSION shRNA Plasmid DNA, Sigma-Aldrich, Merck)
ID: identification; TRC: The RNAi Consortium; UTR: untranslated region; CDS: coding sequence;

## Slide 6
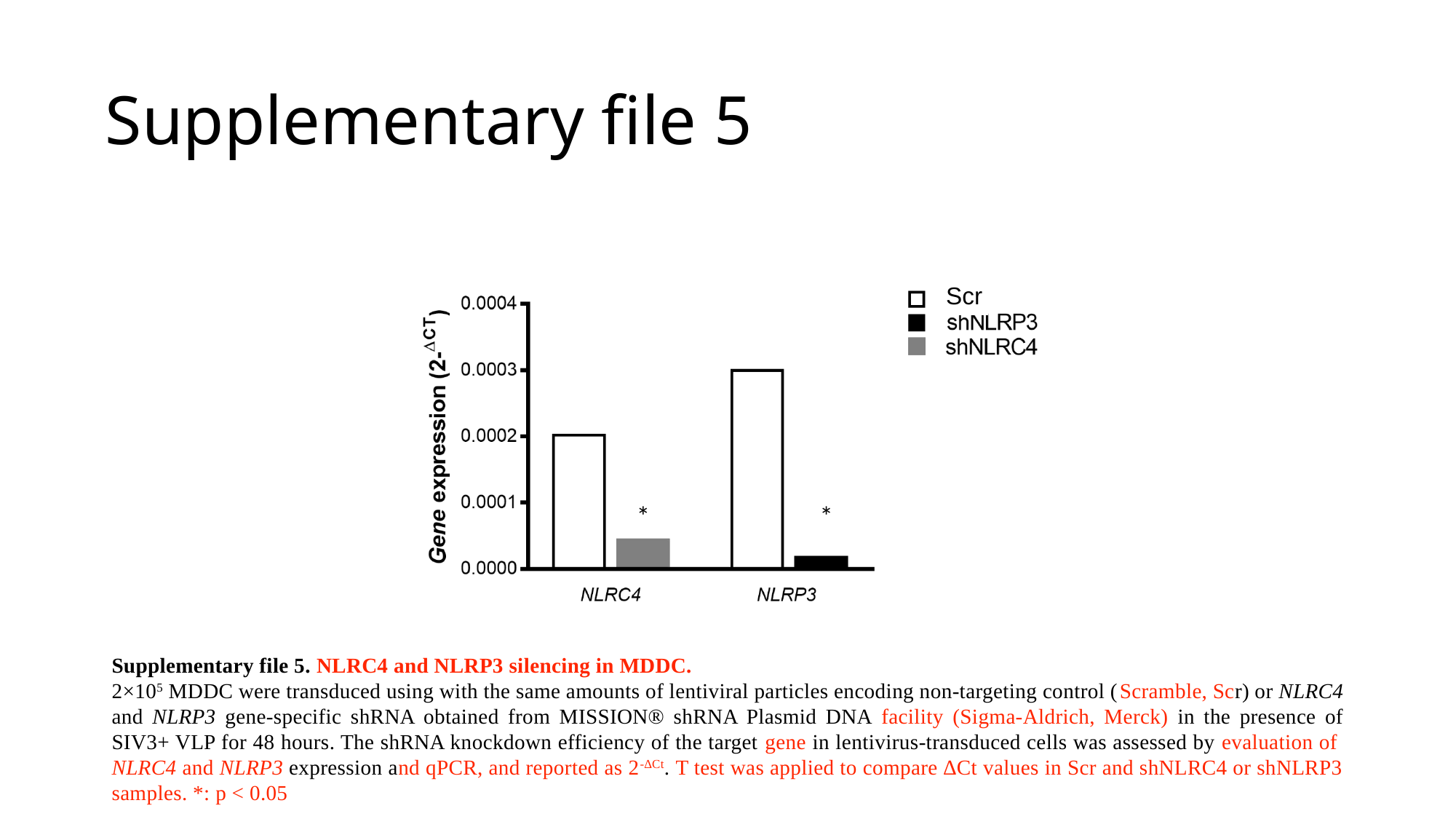

# Supplementary file 5
Scr
*
*
Supplementary file 5. NLRC4 and NLRP3 silencing in MDDC.
2×10^5^ MDDC were transduced using with the same amounts of lentiviral particles encoding non-targeting control (Scramble, Scr) or NLRC4 and NLRP3 gene-specific shRNA obtained from MISSION® shRNA Plasmid DNA facility (Sigma-Aldrich, Merck) in the presence of SIV3+ VLP for 48 hours. The shRNA knockdown efficiency of the target gene in lentivirus-transduced cells was assessed by evaluation of NLRC4 and NLRP3 expression and qPCR, and reported as 2-∆Ct. T test was applied to compare ∆Ct values in Scr and shNLRC4 or shNLRP3 samples. *: p < 0.05

## Slide 7
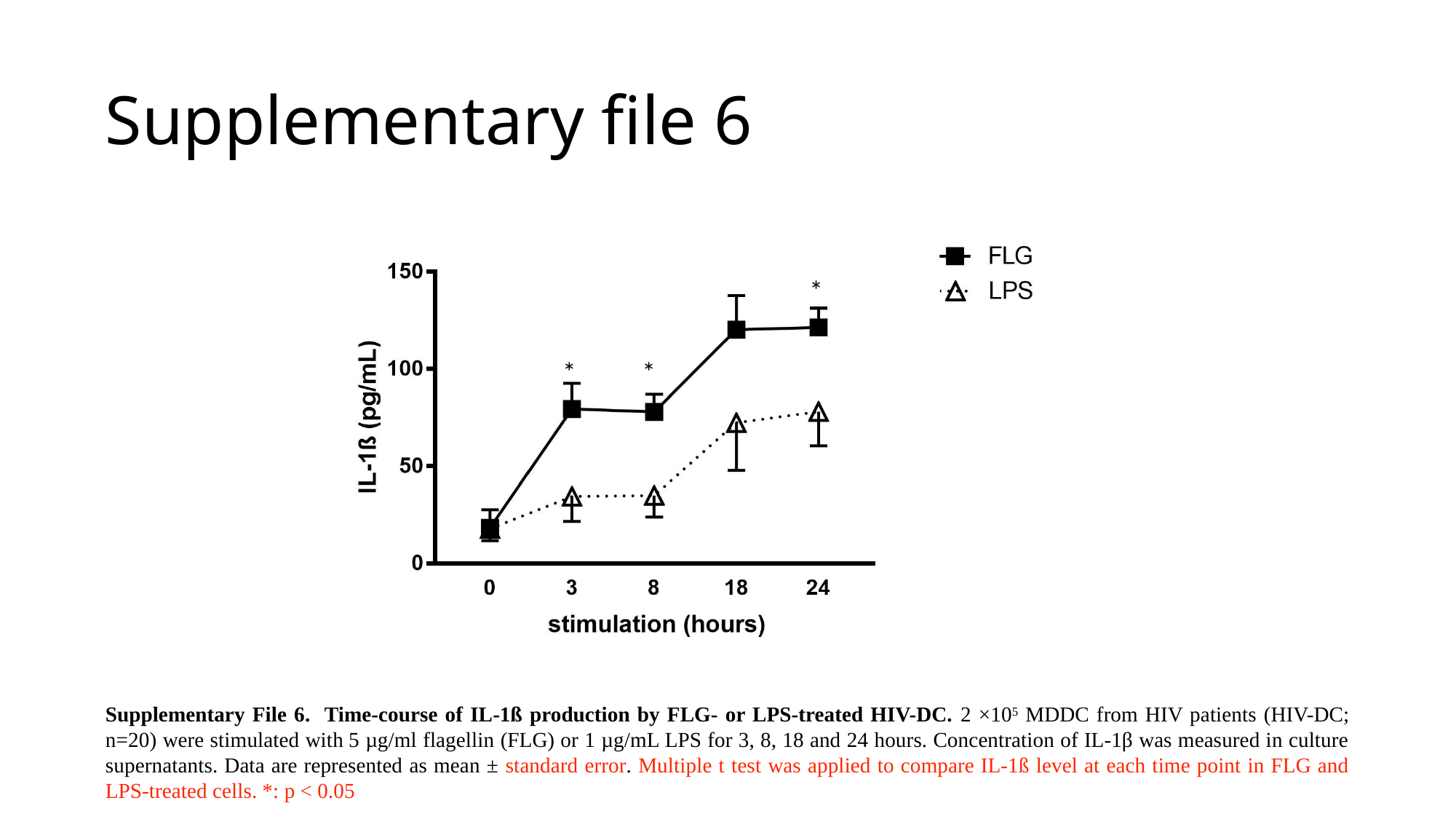

# Supplementary file 6
*
*
*
Supplementary File 6. Time-course of IL-1ß production by FLG- or LPS-treated HIV-DC. 2 ×10^5^ MDDC from HIV patients (HIV-DC; n=20) were stimulated with 5 µg/ml flagellin (FLG) or 1 µg/mL LPS for 3, 8, 18 and 24 hours. Concentration of IL-1β was measured in culture supernatants. Data are represented as mean ± standard error. Multiple t test was applied to compare IL-1ß level at each time point in FLG and LPS-treated cells. *: p < 0.05

## Slide 8
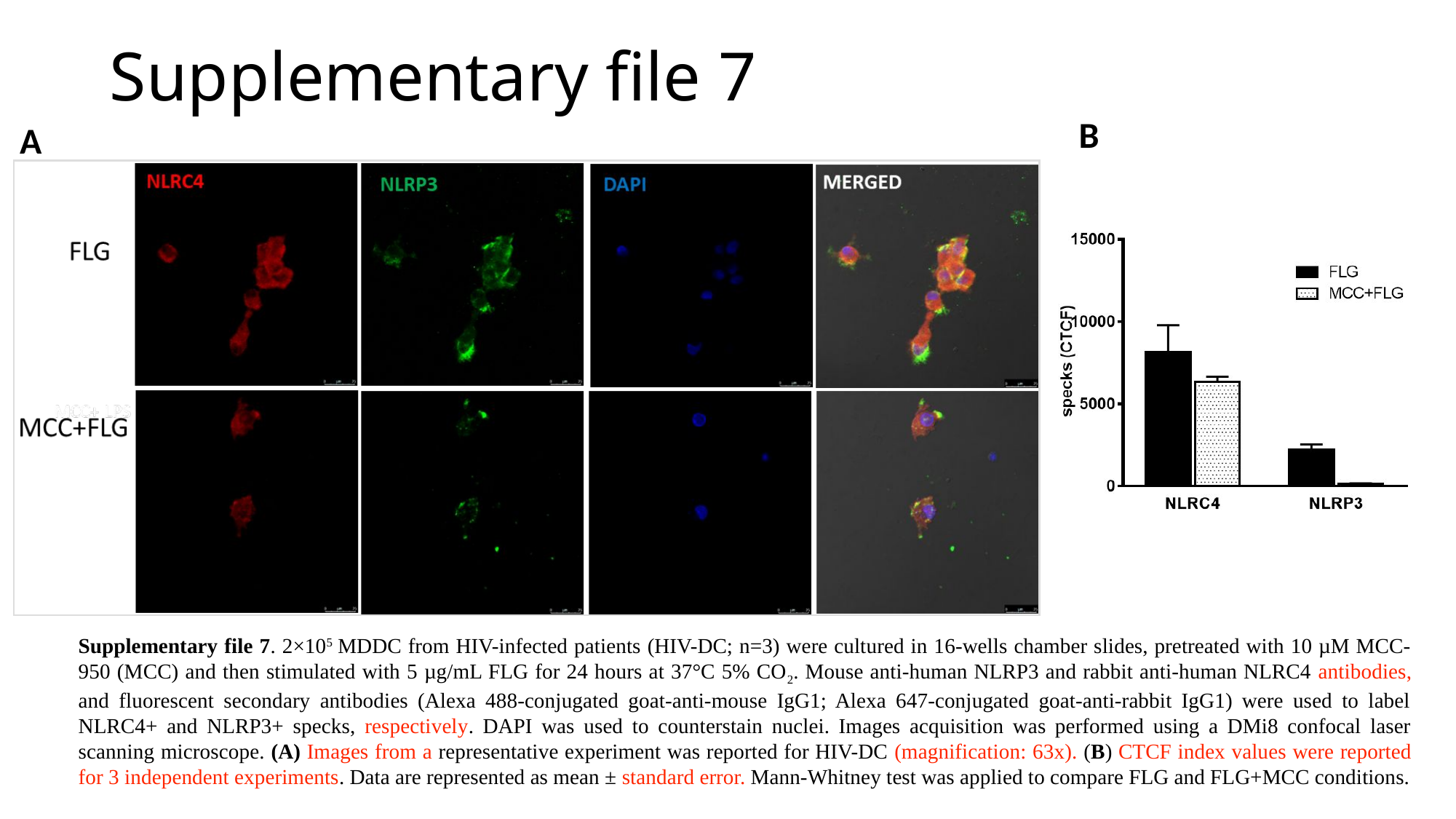

# Supplementary file 7
B
A
Supplementary file 7. 2×10^5^ MDDC from HIV-infected patients (HIV-DC; n=3) were cultured in 16-wells chamber slides, pretreated with 10 µM MCC-950 (MCC) and then stimulated with 5 µg/mL FLG for 24 hours at 37°C 5% CO2. Mouse anti-human NLRP3 and rabbit anti-human NLRC4 antibodies, and fluorescent secondary antibodies (Alexa 488-conjugated goat-anti-mouse IgG1; Alexa 647-conjugated goat-anti-rabbit IgG1) were used to label NLRC4+ and NLRP3+ specks, respectively. DAPI was used to counterstain nuclei. Images acquisition was performed using a DMi8 confocal laser scanning microscope. (A) Images from a representative experiment was reported for HIV-DC (magnification: 63x). (B) CTCF index values were reported for 3 independent experiments. Data are represented as mean ± standard error. Mann-Whitney test was applied to compare FLG and FLG+MCC conditions.
